# Lung type II alveolar epithelial cells collaborate with CCR2^+^ inflammatory monocytes in host defense against poxvirus infection

**DOI:** 10.1038/s41467-022-29308-2

**Published:** 2022-03-29

**Authors:** Ning Yang, Joseph M. Luna, Peihong Dai, Yi Wang, Charles M. Rice, Liang Deng

**Affiliations:** 1 Dermatology Service, Department of Medicine, Memorial Sloan Kettering Cancer Center, New York, NY USA; 2 Laboratory of Virology and Infectious Disease, The Rockefeller University, New York, NY USA; 3 Weill Cornell Medical College, New York, NY USA

**Keywords:** Pattern recognition receptors, Pox virus, Virus-host interactions, Infection, Monocytes and macrophages

## Abstract

The pulmonary immune system consists of a network of tissue-resident cells as well as immune cells that are recruited to the lungs during infection and/or inflammation. How these immune components function during an acute poxvirus infection is not well understood. Intranasal infection of mice with vaccinia virus causes lethal pneumonia and systemic dissemination. Here we report that vaccinia C7 is a crucial virulence factor that blocks activation of the transcription factor IRF3. We provide evidence that type II alveolar epithelial cells (AECIIs) respond to pulmonary infection of vaccinia virus by inducing IFN-β and IFN-stimulated genes via the activation of the MDA5 and STING-mediated nucleic acid-sensing pathways and the type I IFN positive feedback loop. This leads to the recruitment and activation of CCR2^+^ inflammatory monocytes in the infected lungs and subsequent differentiation into Lyve1^−^ interstitial macrophages (Lyve1^−^ IMs), which efficiently engulf viral particles and block viral replication. Our results provide insights into how innate immune sensing of viral infection by lung AECIIs influences the activation and differentiation of CCR2^+^ inflammatory monocytes to defend against pulmonary poxvirus infection.

## Abstract (web summary)

Smallpox is a highly contagious respiratory pathogen associated with a high mortality rate. Here the authors utilize a mouse model of intranasal vaccinia virus infection and show a C7 gene encoded virulence factor attenuates type I IFN release by lung type II alveolar epithelial cells and reduces lung inflammatory monocyte responses.

## Introduction

Host defense against microbial invasion is a highly coordinated process between tissue resident cells and circulating immune cells through recognition of pathogen-associated molecular patterns (PAMPs) by pattern-recognition receptors in resident cells. This in turn results in the production of cytokines that recruit and activate circulating immune cells and amplify effector responses^1^. The pulmonary immune system provides an excellent context to understand such interactions between different compartments and diverse immune cell populations of the lung.

Poxviruses are large cytoplasmic DNA viruses that are important human and veterinary pathogens. Smallpox, a highly contagious infectious disease with a high mortality rate that had claimed hundreds of millions of lives throughout human history, is caused by a human specific poxvirus-- variola virus--through inhalation of airborne droplets^2^. The clinical features include a 10 to 14-day incubation period, followed by an eruption of rash on the oropharynx and skin, accompanied by fever, vomiting, and headaches. 30% of patients contracting Variola major died from a disseminated disease^2^. Smallpox pathogenesis is poorly understood^3^. Moreover, the number of monkeypox cases is increased in recent years, rasing the concern of its outbreak^4,5^. Vaccinia virus Western Reserve (WR) strain has been used widely to investigate poxvirus pathogenesis, and intranasal inoculation in mice results in an acute lung infection followed by virus dissemination to various organs and lethality^6–8^. Therefore, the vaccinia intranasal infection model may shed light on how the lung immune system defends against poxvirus infection and how poxvirus evades such innate immune detection mechanisms.

In the distal airways, alveolar macrophages (AMs) and alveolar epithelial cells (AECs) provide the first barrier against pathogen entry. Upon viral infection, AMs and type II AECs (AECIIs) can produce type I IFNs via nucleic acid-sensing pathways, which sets off an alarm for host defense against viral infection^9,10^. Type I IFN has an important function in host defense against viral infections including vaccinia and many viruses have evolved strategies to evade detection by nucleic acid sensors, to dampen IFN production and signaling^11,12^.

Vaccinia C7 is a host range factor^13^. It is functionally equivalent to another vaccinia host range protein K1 and therefore deletion of both C7L and K1L gene from the vaccinia genome renders the virus replication-incompetent in certain human cells^13^. SAMD9 and WDR6 were identified as host restriction factors for vaccinia virus lacking both the C7L and K1L genes^14^. VACV with both C7L and K1L deletion is highly attenuated in an intranasal infection model, but it gains virulence in *Samd9l*^*−/−*^ mice^15^. The exact mechanism by which C7 attenuates the type I IFN pathway and the function of C7 in vaccinia virus pathogenesis are not well understood.

Here we show that C7 is a virulence factor that attenuates *Ifnb1* gene induction via inhibiting the phosphorylation of interferon regulatory factor 3 (IRF3). A mutant VACV virus with deletion of the C7L gene (VACV∆C7L) is highly attenuated in a mouse intranasal infection model. Upon VACV∆C7L infection, lung AECIIs induce the expression of *Ifnb1* and IFN-stimulated genes (ISGs) via the MDA5 and STING-dependent cytosolic DNA and dsRNA-sensing pathways. IFNAR signaling on AECIIs is important for host control of vaccinia infection and for inducing downstream immune responses. As a result, CCR2^+^ inflammatory monocytes are recruited to the infected lungs and activated to elicit antiviral functions. CCR2^+^ inflammatory monocytes also differentiate into Lyve^−^ interstitial macrophages, which are able to restrict vaccinia replication.

## Results

### A mutant vaccinia virus lacking the C7L gene (VACV∆C7L) is highly attenuated in a mouse intranasal infection model

**Fig. 1:**
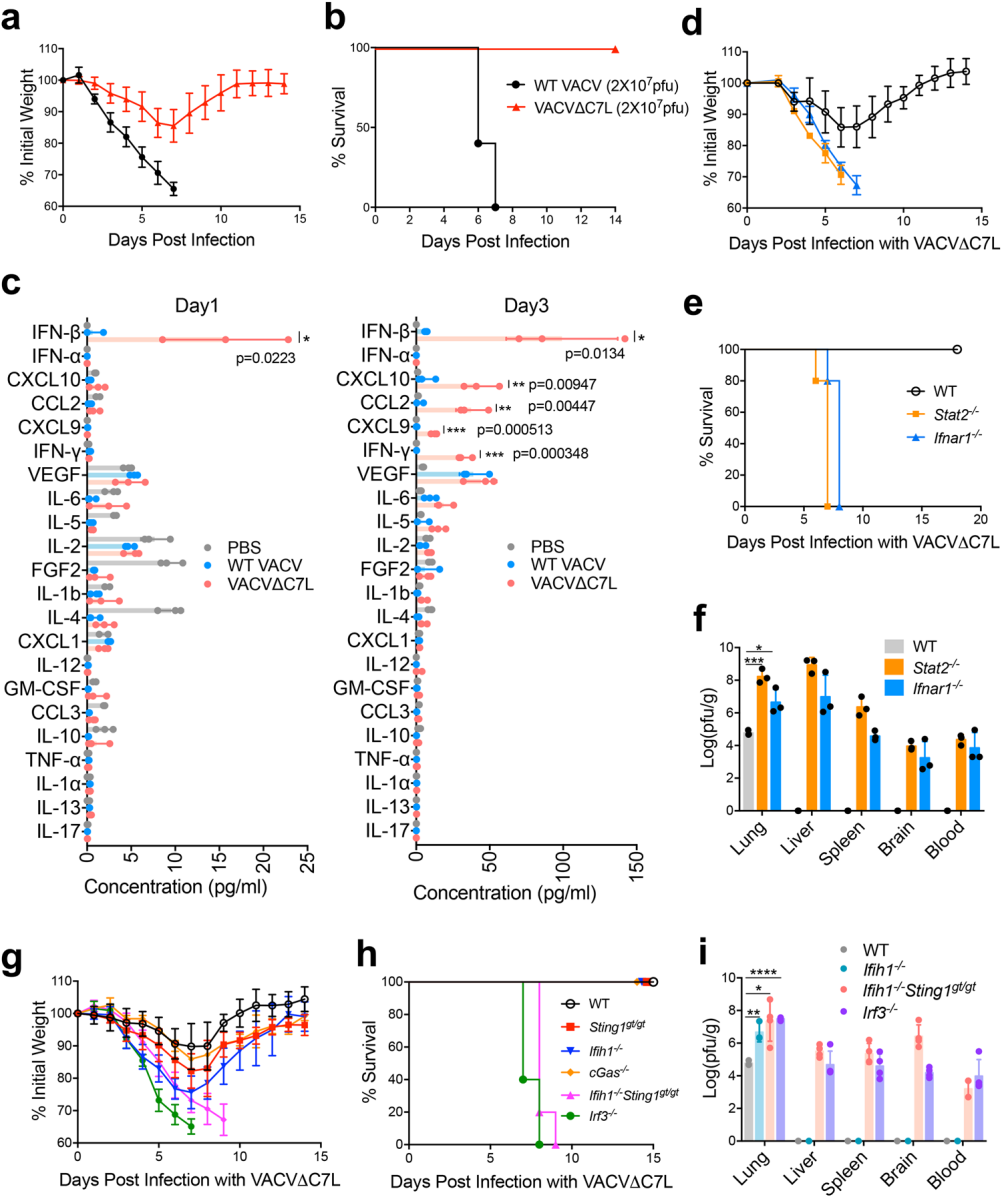
Type I IFN signaling is essential for restricting replication and dissemination of vaccinia virus and C7 protein is a virulence factor for lethal infection. **a**, **b** shown are the percentages of initial weight **a** or Kaplan–Meier survival curve **b** of WT C57BL/6J control mice (*n* = 5 in each group) over days post intranasal infection with WT VACV or VACV∆C7L at a dose of 2 × 10^7^ pfu. **c** Levels of IFN-β and other cytokines/chemokines in BAL from VACV∆C7L or WT VACV (2 × 10^7^ pfu)-infected mice collected at day1 and day 3 post infection determined by ELISA or Luminex. **d**, **e** shown are percentages of initial weight **d** or Kaplan-Meier survival curve **e** over days post intranasal infection with VACV∆C7L at a dose of 2 × 10^7^ pfu in *Stat2*^*−/−*^, *Ifnar1*^*−/−*^, or age-matched WT C57BL/6J control mice (*n* = 5 in each group). **f** Titers of VACV∆C7L in the lungs, livers, spleens, brains and blood of *Stat2*^*−/−*^, *Ifnar1*^*−/−*^, or age-matched WT C57BL/6J control mice at day 4 post intranasal infection with VACV∆C7L at a dose of 2 × 10^7^ pfu. Data are represented as mean ± SD (*n* = 3–5). *p = 0.0148, ***p = 0.000203. **g**, **h** shown are the percentages of initial weight **g** or Kaplan–Meier survival curve **h** over days post intranasal infection with VACV∆C7L at a dose of 2 × 10^7^ pfu in *cGas*^*−/−*^, *Sting*^*gt/gt*^, *Ifih1*^*−/−*^, *Ifih1*^*−/−*^*Sting1*^*gt/gt*^, *Irf3*^*−/−*^, or age-matched WT C57BL/6J control mice (*n* = 5 in each group). **i** Titers of VACV∆C7L in the lungs, livers, spleens, brain and blood of *Ifih1*^*−/−*^, *Ifih1*^*−/−*^*Sting1*^*gt/gt*^, *Irf3*^*−/−*^, or age-matched WT C57BL/6J control mice at day 4 post intranasal infection with VACV∆C7L at a dose of 2 × 10^7^ pfu. *p = 0.0166, **p = 0.0056, ****p = 0.0000008. Data are presented as mean ± SD (*n* = 3-5). *p < 0.05, **p < 0.01, ***p < 0.001 and ****p < 0.0001. Two-tailed unpaired Student’s *t* test was used for comparisons of two groups in the studies. Data are representative of two (**c**, **f**, **i**), or three (**a**, **b**, **d**, **e** and **g**, **h**) independent experiments. Source data are provided as a Source Data file.

To test whether the vaccinia host range protein C7 is a virulence factor, we generated mutant vaccinia (Western Reserve) strain lacking the C7L gene through homologous recombination. The recombinant virus VACV∆C7L, which expresses mCherry or GFP under the vaccinia synthetic early/late promoter, is replication-competent and exhibits similar replication kinetics as WT VACV in BSC40 cells (Supplementary Fig. 1a, b). Intranasal infection of WT VACV infection at 2 × 10^7^ pfu per mouse caused rapid weight loss and 100% lethality (Fig. 1a, b), whereas WT VACV infection at 2 ×10^5^ pfu per mouse resulted in 60% mortality (Supplementary Fig. 1c, d). By contrast, VACV∆C7L infection at 2 ×10^7^ pfu results in less than 20% weight loss, and all of the mice recovered their weight at 11 to 12 days post infection (Fig. 1a, b and Supplementary Fig. 1e, f). These results demonstrate that VACV∆C7L is attenuated by at least 100-fold compared with WT VACV in an intranasal infection model, and therefore C7 is a virulence factor.

### Type I IFN signaling is crucial for host control of VACV∆C7L infection in the lungs

To understand the differences in the lung immune responses to intranasal infection with VACV∆C7L or WT VACV, we isolated the bronchoalveolar lavage fluid (BAL) at day 1 and day 3 post infection and measured the levels of various cytokines and chemokines. At day 1 post infection, VACV∆C7L induced detectable IFN-β level in BAL, whereas WT VACV did not. At day 3 post infection, VACV∆C7L induced much higher levels IFN-β levels in the BAL compared with those collected at day 1 post infection, whereas WT VACV slightly induced IFN-β levels at day 3 post infection (Fig. 1c). Neither WT VACV nor VACV∆C7L induced detectable IFN-α secretion. Proinflammatory cytokines and chemokines analyses in the BAL showed that VACV∆C7L infection resulted in the release of IFN-γ, IL-6, CCL2, CXCL10, and CXCL9 into the BAL (Fig. 1c). The latter four cytokines and chemokines are products of ISGs^16–18^. These results suggest that IFN-β induction in lung tissues upon VACV∆C7L infection is an early event, which can then trigger the activation of down-stream innate immune responses including the expression of ISGs.

We then tested whether type I IFN signaling is important for host defense against VACV∆C7L. We found that the *Stat2*^*−/−*^ and *Ifnar1*^*−/−*^ mice were highly susceptible to VACV∆C7L infection, with rapid weight loss, severe illness, and death (Fig. 1d, e). VACV∆C7L infection of WT mice caused localized infection in the lungs without dissemination of the virus or viremia. VACV∆C7L infection caused higher viral titers in the lungs of *Stat2*^*−/−*^ or *Ifnar1*^*−/−*^ mice compared with those in the WT mice (Fig. 1f). We also observed viremia and dissemination of the virus to various distant organs including the liver, spleen, brain, and blood in *Stat2*^*−/−*^ and *Ifnar1*^*−/−*^ mice (Fig. 1f). The median lethal dose (LD_50_) of VACV∆C7L virus in *Stat2*^*−/−*^ or *Ifnar1*^*−/−*^ mice was also determined and is around 1000 pfu (Supplementary Fig. 1g–j).

### Both the cytosolic dsRNA and DNA-sensing pathways function in restricting pulmonary VACV∆C7L infection

Induction of type I IFN can be mediated by a range of pattern recognition receptors (PRRs)^19^. To test whether the nucleic acid-sensing pathways have a function in host defense against VACV∆C7L infection, we performed intranasal infection of VACV∆C7L in *cGas*^*−/−*^, *Sting1*^*gt/gt*^ (which lack functional STING), *Ifih1*^*−/−*^ (MDA5 knockout), *Ifih1*^*−/−*^*Sting1*^*gt/gt*^ or *Irf3*^*−/−*^ mice. All of the WT, *cGas*^*−/−*^, *Sting1*^*gt/gt*^, or *Ifih1*^*−/−*^ mice lost weight transiently but eventually gained weight and recovered from acute illness. By contrast, all of the *Ifih1*^*−/−*^*Sting1*^*gt/gt*^ or *Irf3*^*−/−*^ mice died at day 7–9 post-infection (Fig. 1g, h). Although viral titers were higher in the infected lungs of *Ifih1*^*−/−*^ mice compared with those in WT mice, VACV∆C7L infection was confined to the lungs in *Ifih1*^*−/−*^ mice. By contrast, in *Ifih1*^*−/−*^*Sting1*^*gt/gt*^ or *Irf3*^*−/−*^ mice infected with VACV∆C7L, systemic dissemination of the virus was observed at day 4 post infection (Fig. 1i). In addition, IFN-β levels in the BAL were abolished in *Irf3*^*−/−*^ mice infected with VACV∆C7L (Supplementary Fig. 1k). Based on these results, we conclude that both the cytosolic dsRNA-sensing pathway mediated by MDA5/IRF3 and the DNA-sensing pathway mediated by cGAS/STING/IRF3 have important functions in host restriction of vaccinia infection in the lungs and in preventing systemic dissemination.

### Lung type II alveolar epithelial cells (ACEIIs) are the major producers of IFN-β in vivo at an early phase of intranasal infection of VACV∆C7L

**Fig. 2:**
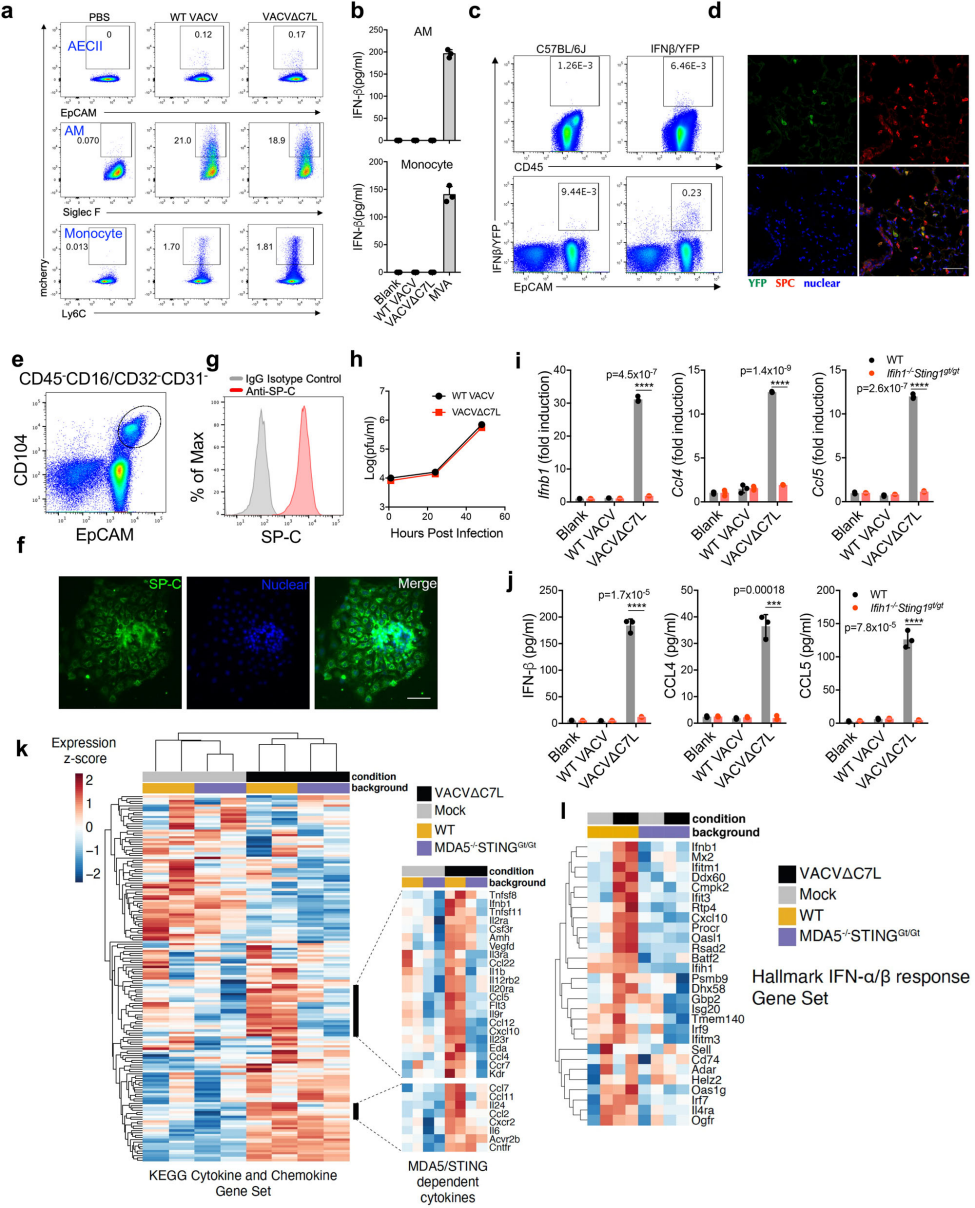
Lung AECIIs produce IFN-β and induces IFN-stimulated genes upon VACV∆C7L infection in a MDA5/STING-dependent manner. **a** Dot plots showing percentages of mCherry-positive cells among AMs (Siglec F^+^CD11c^+^), AECIIs (CD45^−^CD31^−^T1a^−^Sca1^−^EpCAM^+^) and monocytes (Ly6C^+^CD11b^+^) in VACV-mCherry, VACV∆C7L-mCherry-infected lungs from WT C57BL/6J mice at day 1 post infection. **b** ELISA analyses of IFN-β levels in the supernatants of AMs or monocytes isolated from naïve WT C57BL/6J mice infected with viruses at a MOI of 5 for 24 h. **c** Dot plots showing percentages of IFNβ/YFP positive cells among CD45^+^ cells and CD45^−^EpCAM^+^ lung epithelial cells in VACV∆C7L-infected lungs from *Ifnb1*^*Eyfp*^ and WT C57BL/6J mice determined by FACS. **d** Representative confocal images showing expression pattern of IFNβ-secreting cells in lungs from *Ifnb1*^*Eyfp*^ mice infected with VACV∆C7L collected at day 1 post infection. Top left: IFNβ-YFP^+^ cells (green); Top right: surfactant protein C (SPC) positive AECII (red); Bottom left: DAPI staining of nuclei (blue). Scale bar, 50 μm. **e** Gating strategy for the isolation of lineage negative epithelial progenitor cells that are CD45^−^CD16/CD32^−^CD31^−^EpCAM^+^CD104^+^. **f** Cells were cultured in on Matrigel-coated plates as described in methods. The identify of AECII cells were confirmed by SPC^+^ staining. Scale bar, 500 μm. **g** FACS analysis of SPC expression from cultured AECII cells. **h** Viral growth curve in cultured AECII cells at an initial MOI of 0.05. **i** RT-PCR of *Ifnb1*, *Ccl4*, and *Ccl5* gene expression of cultured AECII cells from WT or *Ifih1*^*−/−*^*Sting1*^*gt/gt*^ mice infected with viruses at a MOI of 5 for 12 h. **j** ELISA of IFN-β, CCL4, CCL5 levels in the supernatants of AECII from WT or *Ifih1*^*−/−*^*Sting1*^*gt/gt*^ mice infected with viruses at a MOI of 5 for 24 h. **k** Heatmap of RNAseq showing relative expression of cytokine and chemokines genes between WT AECII and *Ifih1*^*−/−*^*Sting1*^*gt/gt*^ AECII cells. **l** Heatmap of representative type I IFN related differentially expressed genes between WT AECII and *Ifih1*^*−/−*^*Sting1*^*gt/gt*^ AECII cells. Two-tailed unpaired Student’s t test was used for comparisons of two groups in the studies. ***p < 0.001 and ****p < 0.0001. Data are representative of one **k**–**l**, or three **a**–**j** independent experiments and presented as mean ± SD. Source data are provided as a Source Data file.

The lung epithelial cells and alveolar macrophages (AMs) provide the first line defense against pulmonary pathogen infection. FACS analysis of lung tissues harvested after one day post vaccinia virus infection showed that alveolar macrophages, lung epithelial cells and monocytes were infected (Fig. 2a). However, infection of AMs or monocytes isolated from naïve mice with either WT VACV or VACV∆C7L in vitro did not result in IFN-β production. Modified vaccinia virus Ankara (MVA), derived from the vaccinia Ankara strain through more than 500 passages in chicken embryo fibroblasts (CEFs) and lacking many genes involved in immune regulation, was used as a positive control^20^. MVA is non-replicative in myeloid cells. We observed that AMs or monocytes responded to the highly attenuated MVA infection by producing IFN-b (Fig. 2b). To determine which cell population(s) are the major producer(s) of IFN-β upon VACV∆C7L infection, IFN-β-yellow fluorescent protein (YFP) reporter mice (*Ifnb1*^*Eyfp*^) were used to map the cell type(s) responsible for IFN-β production induced by VACV∆C7L infection. We found that the majority of IFN-β/YFP positive cells were CD45^-^EpCAM^+^ lung epithelial cells but not CD45^+^ hematopoietic cells (Fig. 2c).

The EpCAM^+^ lung epithelial cells are mainly composed of two populations, T1a (Podoplanin)^+^ type I alveolar epithelial cells (AECI) and type II alveolar epithelial cells (AECII) (Supplementary Fig. 3a). AECI are large, thin, squamous cells involved in gaseous exchange in the alveolus. AECII are cuboidal, metabolically active cells that contribute to immune defense through the secretion of antimicrobial molecules, immune mediators, and surfactant proteins, and also respond to injury by self-renewal and differentiation into AECI^21–23^. Here we show that the majority of IFN-β secreting CD45^-^EpCAM^+^ lung epithelial cells belonged to AECII cells (Supplementary Fig. 2a). Confocal imaging of lung sections from *Ifnb1*^*Eyfp*^ mice harvested at one day post infection with VACV∆C7L showed IFN-β/YFP-positive cells overlapping with lung AECII marker *surfactant protein C* (*SP-C*) (Fig. 2d). Although our results support that AECIIs are the main responders to VACV∆C7L infection to produce IFN-β, the potential function of AECIs in antiviral innate immunity cannot be completely excluded.

### Primary mouse lung AECIIs induce *Ifnb1*, *Ccl4*, and *Ccl5* gene expression and protein secretion in a MDA5/STING-dependent manner upon VACV∆C7L infection

To firmly establish that the lung AECIIs are capable of producing IFN-β and proinflammatory chemokines upon VACV∆C7L infection and to test whether the induction is dependent on the MDA5 and STING-mediated cytosolic dsRNA and DNA-sensing pathways, we established primary mouse lung AECIIs culture in vitro. The lineage negative epithelial progenitor cells, CD45^−^CD16/CD32^−^CD31^−^EpCAM^+^CD104^+^, were isolated from the lungs of WT and *Ifih1*^*−/−*^*Sting1*^*gt/gt*^ mice (Fig. 2e). The cells were then cultured in vitro to allow their differentiation into AECIIs^24,25^. *SP-C* expression was determined by immunofluorescence staining to confirm AECII identity (Fig. 2f). FACS analysis also showed that the majority of cultured lung AECII express *SP-C* (Fig. 2g). We observed that the replication capacity of VACV∆C7L in primary lung AECII cultured in vitro were similar to that of WT VACV (Fig. 2h). To test the innate immune responses of lung AECIIs to WT VACV or VACV∆C7L infection, AECII from WT and *Ifih1*^*−/−*^*Sting1*^*gt/gt*^ mice were infected with either WT VACV or VACV∆C7L. In WT lung epithelial cells, infection with VACV∆C7L induced higher expression levels of *Ifnb1*, *Ccl4*, and *Ccl5* compared with WT VACV (Fig. 2i). ELISA analysis of supernatants confirmed that VACV∆C7L infection resulted in the secretion of IFN-β, CCL4 and CCL5 from WT AECIIs (Fig. 2j). By contrast, the *Ifih1*^*−/−*^*Sting1*^*gt/gt*^ AECII failed to induce *Ifnb1*, *Ccl4*, and *Ccl5* gene expression and to produce IFN-β, CCL4 and CCL5 upon VACV∆C7L infection (Fig. 2i, j). However, MDA5-deficient AECIIs had modest reduction of *Ifnb1* gene expression and IFN-β secretion compared with WT cells in response to VACV∆C7L; STING-deficient AECII had similar capacity to induce *Ifnb1* in respsone to VACV∆C7L (Supplementary Fig. 2b). These results demonstrate that the induction of *Ifnb1*, *Ccl4*, and *Ccl5* gene expression and protein secretion by VACV∆C7L in AECII is dependent on both MDA5 and STING. There is no detectable *Ifna* gene expression induced upon VACV∆C7L infection in AECIIs (Supplementary Fig. 2c). Most likely it is because of low expression level of Irf7 in AECIIs (Supplementary Fig. 2d). After intranasal inoculation, vaccinia virus may also have contact with bronchiolar epithelium. Clara cells are secretory non-ciliated cells in the tracheal epithelium and bronchioles. We isolated Clara cells (CD45^−^CD16/CD32^−^CD31^−^EpCAM^+^tdTomato^+^) from *Scgb1a1*^*Cre*^*R26*^*tdT*^ mice (Supplementary Fig. 2e). In vitro infection with VACV∆C7L failed to induce *Ifnb1* gene expression in Clara cells (Supplementary Fig. 2f). In addition, VACV∆C7L infection resulted in the generation of dsRNAs in ACEII cells but not in AM cells (Supplementary Fig. 2g). Similar results were obtained by FACS analyses. Both WT VACV and VACV∆C7L infection generated dsRNAs in AECIIs but not in AM cells (Supplementary Fig. 2h). However, only VACV∆C7L infection induced *Ifnb1* gene expression, indicating that C7 is a negative regulator of the cytosolic dsRNA-sensing pathway.

We next performed RNA-seq of VACV∆C7L infected AECII cells from WT or *Ifih1*^*−/−*^*Sting1*^*gt/gt*^ mice to determine the global transcriptional response to infection. We confirmed a MDA5/STING-dependent upregulation of cytokine and chemokine genes after VACV∆C7L infection in AECII cells, including *Ifnb1*, *Ccl2*, *Ccl7*, *Ccl4*, *Ccl5*, and *Cxcl10* (Fig. 2k). The expression of a number of genes within the IFN-α/β response Gene Set were MDA5/STING- dependent, such as *Dhx58*, *Oasl1*, *Rsad2*, *Ifit3*, *Ifitm1*, and *Mx2* (Fig. 2i).

We reasoned that if the inability to induce IFN-β from lung AECII by WT VACV is the main contributing factor for its virulence, intranasal administration of IFN-β might be able to rescue mice from lethal infection with WT VACV. IFN-β treatment initiated one day after WT VACV infection at 2 ×10^6^ pfu successfully delayed weight loss and protected mice from lethality (Supplementary Fig. 2i, j). Taken together, our results confirm that IFN-β production and signaling in the lungs are critical for host defense against vaccinia virus infection.

### Vaccinia C7 inhibits IFNB gene induction by innate immune pathways and type I IFN signaling

**Fig. 3:**
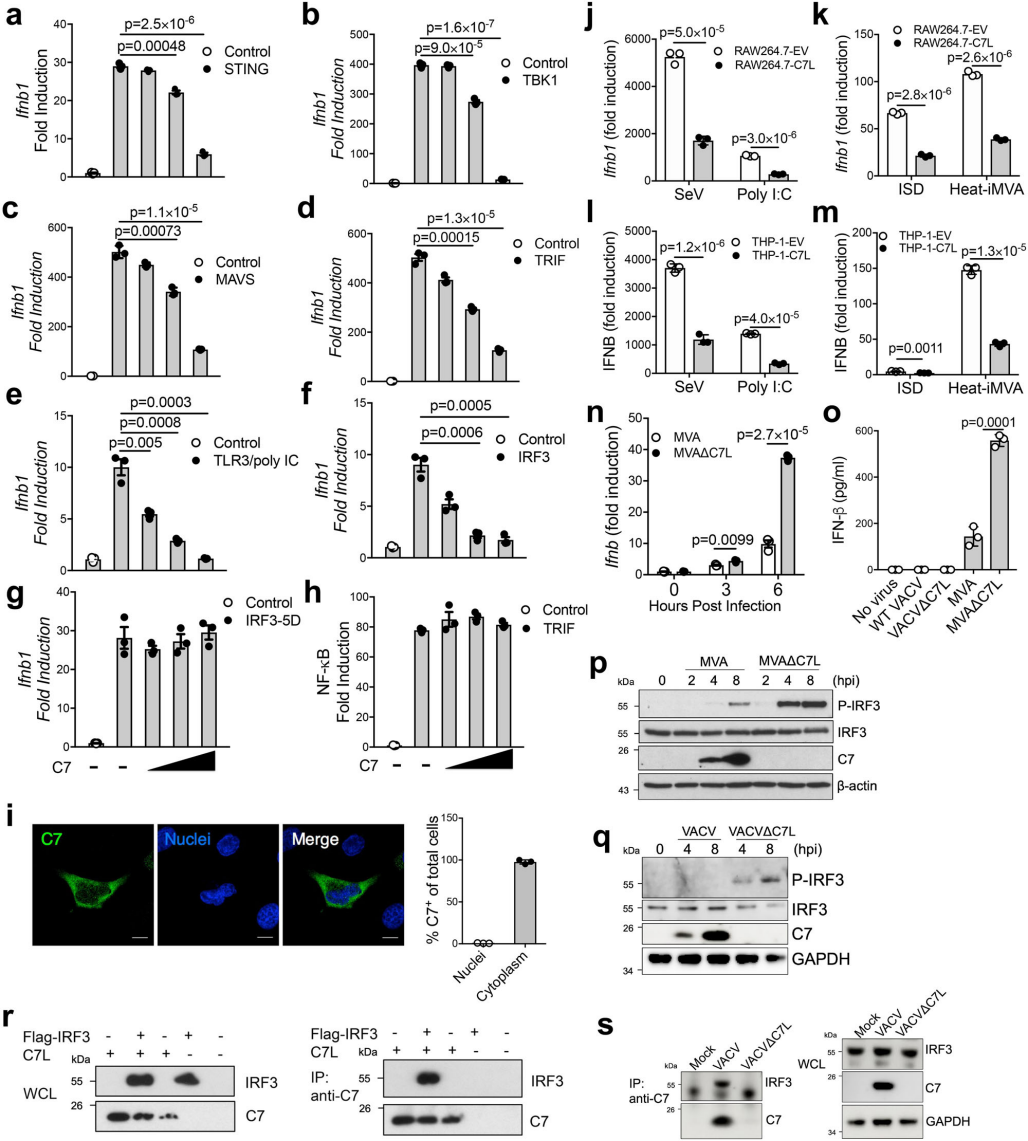
Vaccinia C7 inhibits *Ifnb1* gene induction by interacting with IRF3 and preventing IRF3 phosphorylation. Dual-luciferase assay of HEK293T cells transfected with *Ifnb1*-firefly luciferase reporter, pRL-TK expressing *Renilla* luciferase, vaccinia C7L-expressing or control plasmid, and STING **a**, TBK1 **b**, MAVS **c**, TRIF **d**, IRF3 **f**, IRF3-5D **g**. Cells were harvested at 24 h post transfection. **e** TLR3-expressing HEK293T cells were treated with poly I:C for 24 h. **h** Luciferase assay of HEK293T cells transfected with NF-kB-firefly luciferase reporter and TRIF-expressing plasmid. **i** Left: Representative confocal images showing expression pattern of C7 in Hela cells after vaccinia virus infection. Scale bar, 10 μm. Right: Quantitation of C7^+^ cells in over 100 cells. **j**–**m** RAW264.7 cell expressing vaccinia C7 (RAW264.7-C7L) or with empty vector (RAW264.7-EV) **j**, **k** or THP-1 cell expressing vaccinia C7 (THP-1-C7L) or with empty vector (THP-1-EV) **l**–**m** were infected with Sendai virus (SeV), heat-inactivated MVA (Heat-iMVA) at a MOI of 5, transfected with poly I:C or interferon stimulatory DNA (ISD). 24 h later. *Ifnb1* gene expression level was measured by RT-PCR. **n** Bone marrow-derived dendritic cells (BMDCs) were infected with either MVA or MVA∆C7L at a MOI of 5. *Ifnb1* gene expression levels were determined by RT-PCR. **o** BMDCs were infected with viruses at a MOI of 5, IFN-β levels in the supernatants were determined by ELISA. **p** Immunoblot analysis of cell lysates from virus infected BMDCs (MOI = 5.0). **q** Immunoblot analysis of cell lysates from virus infected AECII cells (MOI = 5.0). **r** HEK293T cells were co-transfected with Flag-tagged IRF3 or C7L either alone or in combination. Whole cell lysates were immunoprecipitated with anti-C7 antibody and immunoblotted with anti-Flag antibody. **s** AECII cells were infected with viruses at a MOI of 5 for 6 h. Whole cell lysates were immunoprecipitated with anti-C7 antibody and immunoblotted with anti-IRF3 antibody. Two-tailed unpaired Student’s t test was used for comparisons of two groups in the studies. Data are representative of two **i**–**s** or three **a**–**h** independent experiments and presented as mean ± SD. Source data are provided as a Source Data file.

To understand the molecular mechanism by which vaccinia C7 antagonizes the IFN pathway, we utilized a dual-luciferase assay system to evaluate the function of vaccinia C7 in the regulation of STING, TBK1, MAVS, TRIF, TLR3, or IRF3-induced IFNB promoter activation. Overexpression of STING resulted in a 30-fold induction of IFNB promoter activity compared with that in the control sample without STING. Co-transfection of increasing amounts of C7L expression plasmid led to a significant reduction of STING-induced IFNB promoter activity (Fig. 3a). Similarly, co-transfection of increasing amounts of C7L expression plasmid led to an over 90% reduction of TBK1-induced IFNB promoter activity (Fig. 3b). The TBK1-IRF3 axis is important for signal transduction in several pathways, including cGAS-cGAMP-STING, RIG-I/MDA5-MAVS, TLR3-TRIF. MAVS or TRIF overexpression resulted in elevated IFNB promoter activity compared to the control, wherein C7 blocked either MAVS or TRIF-induced luciferase signal by 70% (Fig. 3c, d). Treatment with poly I:C in TRL3 overexpressing cells promoted induction of Ifnb promoter activity and IFNB promoter signal was reduced with increasing C7 (Fig. 3e). These results indicate that C7 exerts an inhibitory effect on STING, MAVS, TLR3/poly (I:C), TRIF, and TBK1-induced IFNB promoter activity. IRF3 is an essential transcription factor for the IFNB promoter and over-expression of C7 caused a 70% reduction of IRF3-induced IFNB promoter activity (Fig. 3f), whereas overexpression of C7 failed to reduce IRF3-5D-induced IFNB promoter activity (Fig. 3g). IRF3-5D is a constitutively active, phosphorylation-mimetic mutation of IRF3^26^. In addition, C7 does not affect NF-κB gene activation induced by TRIF overexpression (Fig. 3h). Taken together, our results suggest C7 may target IRF3 activation at the level of phosphorylation.

C7 is detected in the cytoplasm after vaccinia virus infection (Fig. 3i). To assess the effect of vaccinia C7 in *Ifnb1* gene induction in immune cells, we generated cell lines stably expressing vaccinia C7 or empty vector as control, including mouse macrophage RAW264.7 and human THP-1. SeV infection or poly (I:C) treatment induced *Ifnb1* gene expression in both RAW264.7 and THP-1 cells via the activation of cytosolic RNA-sensing pathway, which was reduced in the respective C7-overexpressing cell lines (Fig. 3j, l). Similarly, vaccinia C7 reduced ISD or Heat-iMVA-induced *Ifnb1* gene expression in RAW264.7 or THP-1 cells, demonstrating that C7 also blocked the activation of the cytosolic DNA-sensing pathway (Fig. 3k, m). Similar to infection in lung alveolar macrophages, MVA∆C7L induced higher levels of *Ifnb1* gene expression in BMDCs compared with MVA (Fig. 3n). Whereas neither WT VACV nor VACV∆C7L induced IFN-β secretion in BMDCs, both MVA and MVA∆C7L infection triggered IFN-β production, with MVA∆C7L inducing higher levels of IFN-β secretion in BMDCs compared with MVA (Fig. 3o). MVA∆C7L infection of BMDCs induced higher levels of phosphorylation of IRF3 compared with MVA (Fig. 3p). VACV∆C7L infection of lung AECIIs induced phosphorylation of IRF3 while WT VACV failed to induce (Fig. 3q). Taken together, these results indicate that C7 blocks IRF3 phosphorylation in lung AECIIs.

To probe the mechanisms by which vaccinia C7 exerts its inhibitory effects on IRF3 phosphorylation, a co-immunoprecipitation assay was performed to determine whether vaccinia C7 interacts with IRF3. HEK293T cells were co-transfected with Flag-tagged IRF3 or C7 either alone or in combination. Whole cell lysates (WCL) showed the expression of IRF3 and C7 in transfected cells (Fig. 3r). Following immunoprecipitation of the whole cell lysates with an anti-C7 antibody, we observed that the Flag-tagged IRF3 was pulled down by the anti-C7 antibody from whole cell lysates (Fig. 3r). Furthermore, co-immunoprecipitation assay of WT VACV infected lung AECII cell lysates with anti-C7 antibody showed that C7 protein interacted with endogenous IRF3 (Fig. 3s). Our results demonstrate that C7 interacts with IRF3, which may contribute to its inhibitory effects on IFN gene induction.

### Type I IFN signaling on lung AECIIs cells have a critical function in host defense against VACV∆C7L infection

**Fig. 4:**
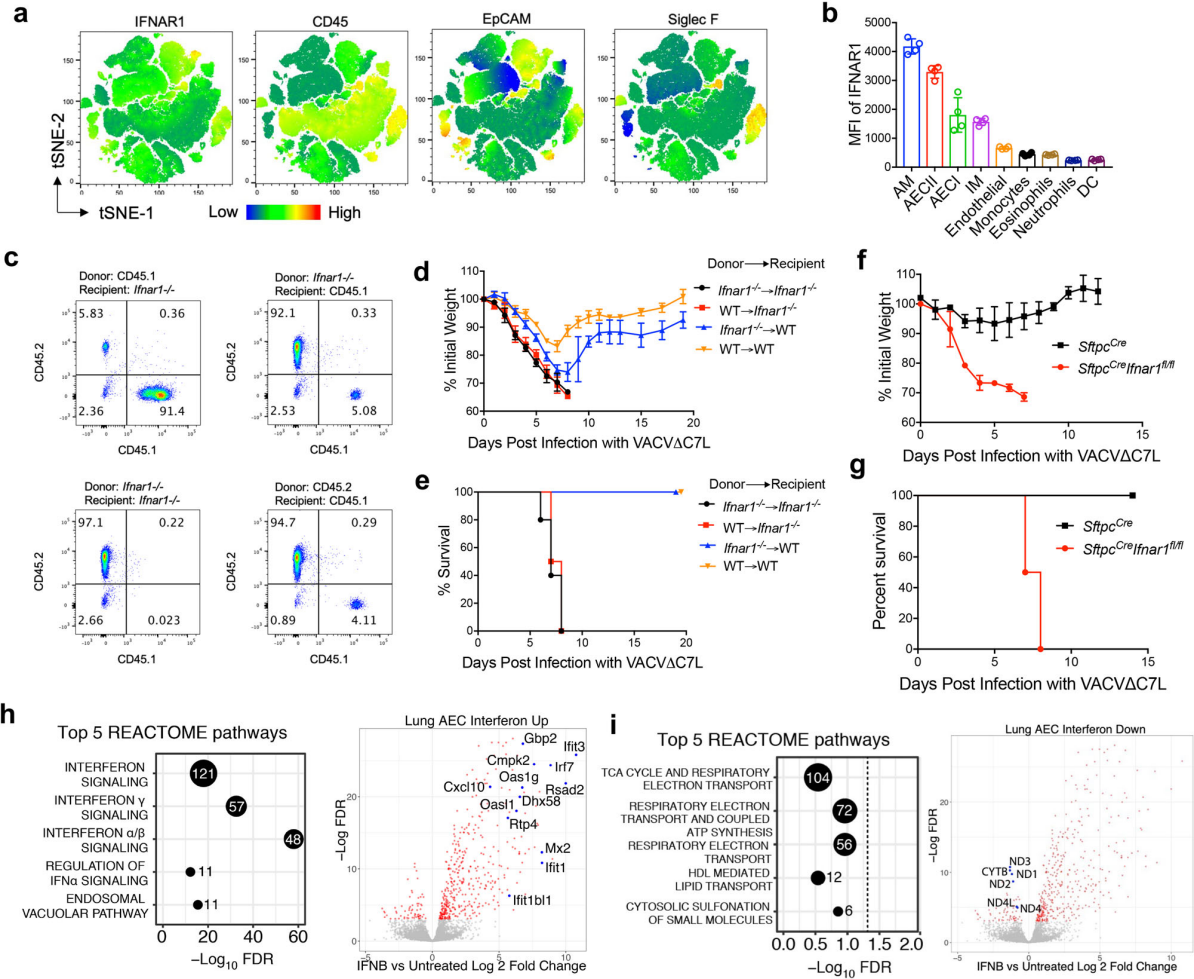
Type I IFN signaling in lung AECIIs plays a crucial function in host restriction of vaccinia infection. **a**, **b** FACS analysis of IFNAR1 expression in different cell populations from lungs in C57BL/6J mice. **a** t-SNE analysis showing different cellular markers-IFNAR1, CD45, EpCAM and Siglec F expression in different cell populations from lungs. (b) MFI of IFNAR1 expression in different cell populations from lungs. **c** shown are the reconstitution efficiency in bone marrow chimeras (*Ifnar1*^*−/−*^*→ Ifnar1*^*−/−*^, WT → WT, *Ifnar1*^*−/−*^→ WT, and WT→ *Ifnar1*^*−/−*^). **d**, **e** shown are the percentages of initial weight **d** or Kaplan–Meier survival curve **e** over days post intranasal infection with VACV∆C7L at 2 × 10^7^ pfu in bone marrow chimeras (*Ifnar1*^*−/−*^*→ Ifnar1*^*−/−*^, WT → WT, *Ifnar1*^*−/−*^→ WT, and WT→ *Ifnar1*^*−/−*^) (*n* = 5 in each group). **f**, **g** shown are the percentages of initial weight **f** or Kaplan-Meier survival **g** over days post intranasal infection with VACV∆C7L at 2 × 10^7^ pfu in *Sftpc*^*cre*^*Ifnar1*^*fl/fl*^ and *Sftpc*^*cre*^ mice after treatment of tamoxifen (*n* = 5 in each group). **h**, **i** Significance dotplots and volcano plots depicting top differentially expressed pathways (left) and genes (right), respectively, between naïve and IFN-β (500 U/ml) treated AECII cells from C57BL/6J mice. Dashed line indicates a false discovery rate cutoff of 0.05. Differentially expressed genes are highlighted in red. Data are presented as mean ± SD. Data are representative of one **h**, **i** or two **a**–**g** independent experiments. Source data are provided as a Source Data file.

To determine which cell population(s) IFN-β may act on to defend against VACV∆C7L infection, we first analyzed IFNAR1 protein expression in different cell populations from lung (Supplementary Fig. 3a, b). We found that IFNAR1 is highly expressed in AECII in addition to AMs and interstitial macrophages (IMs) (Fig. 4a, b). To distinguish the contributions of IFNAR signaling in hematopoietic cells vs. non-hematopoietic cells to host restriction of VACV∆C7L infection in the lungs, we generated bone marrow chimeras. Analysis of CD45.1 and CD45.2 markers of immune cells in the bone marrow chimeras showed the desired reconstitution of hematopoietic cells in the blood (Fig. 4c). Consistent with previous results, VACV∆C7L infection in WT → WT mice resulted in transient weight loss and all of the mice survived the infection. By contrast, VACV∆C7L infection in *Ifnar1*^*−/−*^ → *Ifnar1*^*−/−*^ mice resulted in rapid weight loss and 100% mortality (Fig. 4d, e). All of the WT recipient mice reconstituted with *Ifnar1*^*−/−*^ bone marrow cells survived despite losing more weight compared with WT recipient mice reconstituted with WT bone marrow cells (Fig. 4d, e). This suggests that type I IFN signaling on hematopoietic resident cells contribute to host defense against VACV∆C7L but to a limited extent. By contrast, all of the *Ifnar1*^*−/−*^ recipient mice reconstituted with WT bone marrow cells succumbed to VACV∆C7L infection, indicating that type I IFN signaling on non-hematopoietic resident cells is indispensable for host restriction of VACV∆C7L infection (Fig. 4d, e).

Given that lung AECIIs are the major early producers of IFN-β upon intranasal VACV∆C7L infection and highly IFNAR1 expressed, we hypothesized that IFN signaling on lung AECII might be important for restricting VACV∆C7L infection in the lungs. To test that, we used *Sftpc*^*cre*^*Ifnar1*^*fl/fl*^ mice lacking IFNAR1 protein specifically in lung AECII upon tamoxifen-induced cre expression^27^, which was confirmed in our analysis (Supplementary Fig. 3c). Whereas the control mice had mild transient weight loss upon VACV∆C7L infection, all of the *Sftpc*^*cre*^*Ifnar1*^*fl/fl*^ mice suffered severe weight loss and were euthanized at day 7 or 8 post infection when they lost more than 30% of their original weight (Fig. 4f, g). These results demonstrate that type I IFN signaling in lung AECII contributes to restricting viral infection in the lungs.

To further elucidating type I IFN signaling on lung AECIIs after IFN-β production, we performed transcriptomic profiling of lung AECII isolated from WT C57BL/6J mice with or without IFN-β treatment. IFN-β treated AECIIs displayed elevated interferon signaling activation (Fig. 4h), including many MDA5/STING-dependent ISGs as described in Fig. 2L. For example, *Gbp2*, *cmpk2*, *Ifit3*, *Ifitm1*, *Dhx58*, *Oas1*, *Rsad2*, *Irf7*, *Mx2*, *Cxcl10*, and others (Fig. 4h). IFN-β treatment also resulted in a down-regulation of several pathways, including tricarboxylic acid cycle (TCA) cycle, respiratory electron transport and coupled ATP synthesis (Fig. 4i).

### Intranasal infection of VACV∆C7L results in the influx of dendritic cells (DCs), monocytes, neutrophils, CD8^+^, and CD4^+^ T cells into bronchoalveolar space of the infected lungs

To understand the reduced virulence of VACV∆C7L compared with WT VACV in the intranasal infection model, we performed immune cell analyses of BAL from WT VACV or VACV∆C7L-infected mice. Lung resident AMs comprise the majority of CD45^+^ cells in the BAL in the PBS mock-infected mice. WT VACV infection resulted in slight reduction of the percentage of Siglec F^+^CD11c^+^ alveolar macrophages at day 5 post infection, with a mild increase of other myeloid cell populations in the BAL compared with mock-infected controls (Supplementary Fig. 4a-e). By contrast, VACV∆C7L infection caused influx of myeloid cells, which included Ly6C^+^CD11b^+^ inflammatory monocytes, Ly6G^+^ neutrophils, and MHCII^+^CD11c^+^ DCs, into bronchoalveolar space at day 5 post infection (Supplementary Fig. 4a-e). Furthermore, VACV∆C7L infection caused marked increase of CD4^+^ and CD8^+^ T cells in the BAL compared with WT VACV or PBS mock infection control. CD8^+^ and CD4^+^ T cells comprised of 40% and 10% of CD45^+^ cells in BAL after VACV∆C7L infection (Supplementary Fig. 4f-i). Vaccinia virus B8R_20-27_ is an immunodominant peptide^28^. Viral-specific IFN-γ^+^CD8^+^ T cells were detected in BAL after stimulation with B8R epitope TSYKFESV, but not with irrelevant epitope- SIINFEKL (Supplementary Fig. 4j, k).

### CCR2^+^ inflammatory monocytes are essential for host restriction of VACV∆C7L infection in the lungs

**Fig. 5:**
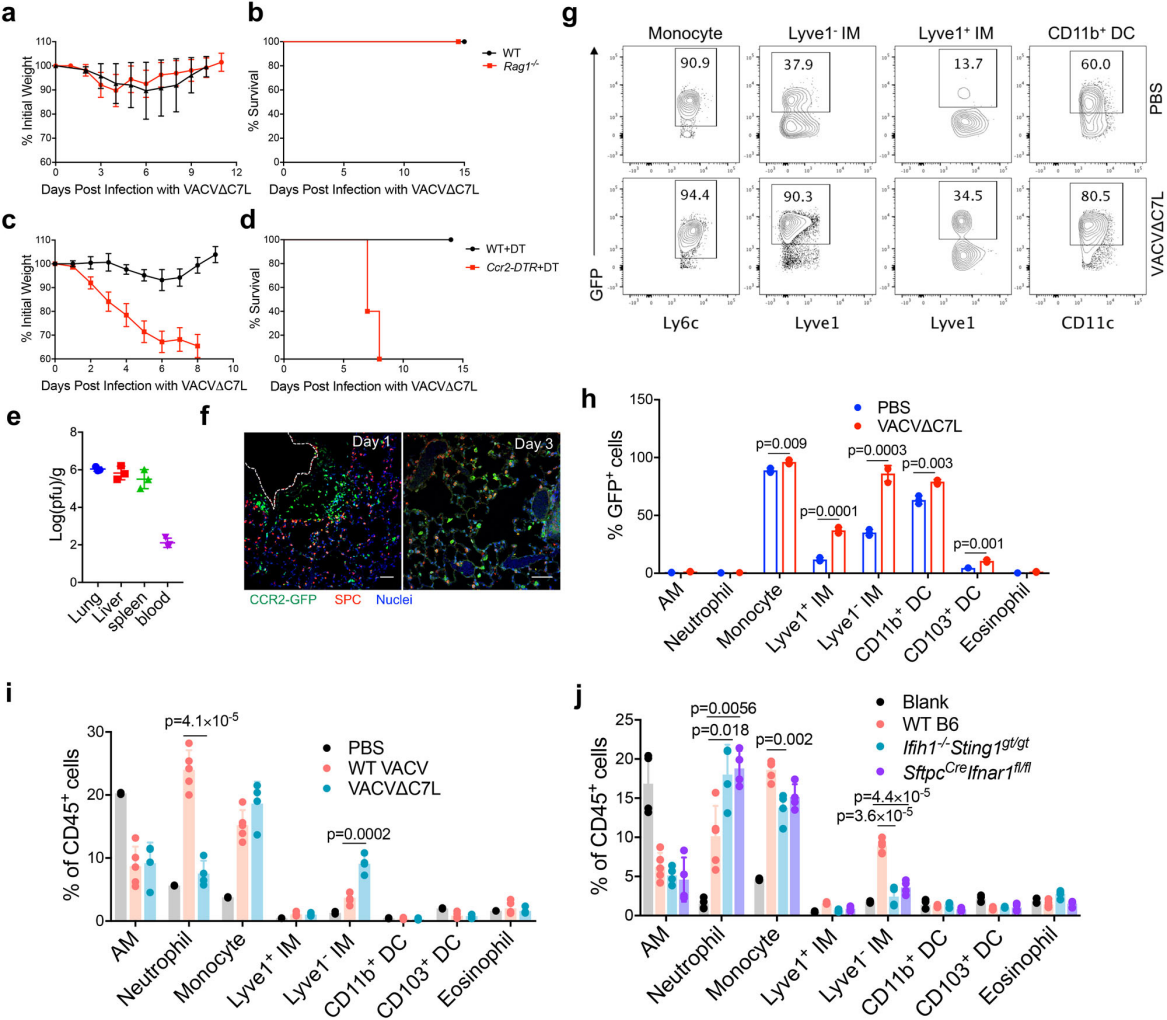
CCR2^+^ inflammatory monocytes and its derived Lyve1^-^ interstitial macrophages function in restricting VACV∆C7L infection. **a**, **b** shown are the percentages of initial weight **a** or Kaplan–Meier survival curve **b** over days post intranasal infection with VACV∆C7L at 2 × 10^7^ pfu in *Rag1*^*−/−*^ and age-matched WT C57BL/6J mice. (*n* = 5 in each group). **c**, **d** shown are the percentages of initial weight **c** or Kaplan-Meier survival curve **d** over days post intranasal infection with VACV∆C7L at 2 × 10^7^ pfu in *Ccr2-DTR* and age-matched WT C57BL/6J mice treated with DT (*n* = 5 in each group). **e** Titers of VACV∆C7L in the lungs, livers, spleens, and blood of *Ccr2-DTR* mice mice treated with DT at day 4 post intranasal infection with VACV∆C7L at a dose of 2 × 10^7^ pfu. **f** Representative confocal images showing location of CCR2^+^ cells in adult lungs from *Ccr2-GFP* mice infected with VACV∆C7L at 2 ×10^7^ pfu collected at day 1 or day 3 post infection. Scale bar, 50 μm. **g**, **h** Representative flow cytometry dot plots **g** and bar graph **h** showing an increase of GFP^+^ Lyve1^-^ IMs, GFP^+^Lyve1^+^ IMs, GFP^+^Ly6C^+^ monocytes and GFP^+^MHCII^+^CD11C^+^ DCs in the lungs of *Ccr2-GFP* mice at day 3 post infection with VACV∆C7L at 2 × 10^7^ pfu compared with PBS-mock infected mice (*n* = 3 in each group). **i** Flow cytometry analysis showing changes of myeloid cells in the lungs of C57BL/6J mice at day 3 post infection with WT VACV, VACV∆C7L at 2 × 10^7^ pfu or PBS-mock infected mice (*n* = 2–5 in each group). **j** Flow cytometry analysis showing changes of myeloid cells in the lungs of WT C57BL/6J, *Ifih1*^*−/−*^*Sting1*^*gt/gt*^ or *Sftpc*^*cre*^*Ifnar1*^*fl/fl*^ mice at day 3 post infection with VACV∆C7L at 2 × 10^7^ pfu. PBS was used as a mock infection blank control in WT mice (*n* = 4–5 in each group). Two-tailed unpaired Student’s *t* test was used for comparisons of two groups in the studies. Data are representative of two (**a**-**b**, **f**–**h**) or three (**c**–**e**, **i**–**j**) independent experiments and presented as mean ± SD. Source data are provided as a Source Data file.

To establish the immune cell population(s) involved in restricting VACV∆C7L infection, we first performed intranasal infection of VACV∆C7L in WT and *Rag1*^*−/−*^ mice, which lack mature T and B cells. VACV∆C7L infection resulted in only mild weight loss in both WT and *Rag1*^*−/−*^ mice and all of the mice recovered their weight around day 10 and 11 post infection and survived (Fig. 5a, b). Second, antibody depletion of NK cells or depletion of AMs with intranasal application of liposomal clodronate did not affect VACV∆C7L-induced weight loss and did not enhance mortality (Supplementary Fig. 5a–c). These results indicate that T, B, NK, and AMs are not important for controlling VACV∆C7L pulmonary infection in this intranasal infection model. Third, we used *Ccr2-DTR* mice to transiently deplete CCR2^+^ monocytes prior to VACV∆C7L infection by administering diphtheria toxin (DT) intraperitoneally^29^. *Ccr2-DTR* mice treated with DT were much more susceptible to VACV∆C7L infection compared with WT mice treated with DT, with more rapid weight loss and 100% mortality at day 7 or 8 post infection (Fig. 5c, d). Depletion of CCR2^+^ monocytes also resulted in viremia and systemic dissemination (Fig. 5e). These results demonstrate that CCR2^+^ inflammatory monocytes have an important function in controlling vaccinia infection in the lungs and in preventing systemic dissemination.

To track the dynamics of CCR2^+^ monocytes after intranasal viral infection, we used *Ccr2-GFP* reporter mice in which enhanced GFP is expressed in CCR2^+^ cells under the control of CCR2 promoter^29^. One day after intranasal infection with VACV∆C7L, we observed that CCR2-GFP^+^ monocytes were recruited into interstitial space between blood vessel and alveolar structure. However, at day three post infection, the CCR2-GFP^+^ monocytes were diffusely localized in the alveolar area (Fig. 5f). In infected tissues, monocytes may differentiate into tissue macrophages and dendritic cells. It has been recently shown that CCR2^+^Ly6C^+^ monocytes can differentiate into two distinct IM populations, Lyve1^lo^MHCII^hi^ and Lyve1^hi^MHCII^lo^ in the lungs^30,31^. Here we investigated the fate of CCR2^+^Ly6C^+^ monocytes in the lungs from mice infected with VACV∆C7L by tracing CCR2-GFP expression in myeloid cells. Intranasal infection of WT mice with VAC∆C7L leads to a marked increase of IMs (especially Lyve1^−^ IMs), and a modest increase of CD11b^+^ DCs in the infected lungs (Fig. 5g, h). The percentages of GFP^+^ cells were low in other myeloid cells, including alveolar macrophages, CD103^+^ DC, neutrophils and eosinophils (Supplementary Fig. 5d). Taken together, our results indicate that intranasal infection of VACV∆C7L leads to the recruitment of CCR2^+^ monocytes into the lungs, which can further differentiate into Lyve1^−^ IMs and DCs under the influence of an inflammatory milieu in the infected lungs.

### The MDA5 and STING-mediated nucleic acid-sensing pathway and IFNAR feedback loop in the lung AECIIs contribute to the generation of Lyve1^−^ IMs after VACV∆C7L infection

To understand the potential contribution of inflammatory monocytes and its derived cells against vaccinia virus infection, we first compared the myeloid cell populations in the lungs at day three post infection with either WT VACV or VACV∆C7L. We observed higher percentages of Lyve1^−^ IMs in VACV∆C7L-infected lungs compared with those infected with WT VACV (Fig. 5i). By contrast, WT VACV infection resulted in higher percentages of neutrophils in the infected lungs compared with VACV∆C7L, most likely because of severe lung injury (Fig. 5i and Supplementary Fig. 5e). These results suggest that IFN induction and signaling in the lung AECs might contribute to the Lyve1^−^ IMs development. To test this, we performed intranasal infection of VACV∆C7L in WT, *Ifih1*^*−/−*^*Sting1*^*gt/gt*^, and *Sftpc*^*cre*^*Ifnar1*^*fl/fl*^ mice. We found that intranasal infection of VACV∆C7L resulted in the generation of Lyve1^−^ IMs in WT mice, which were markedly reduced in *Ifih1*^*−/−*^*Sting1*^*gt/gt*^ or *Sftpc*^*cre*^*Ifnar1*^*fl/fl*^ mice (Fig. 5j and Supplementary Fig. 5f). These results indicate that IFN-β production mediated by the cytosolic DNA and dsRNA-sensing pathways and type I IFN signaling in AECIIs are critical for the differentiation into Lyve1^−^ IMs from CCR2^+^ monocytes.

### CCR2^+^Ly6C^+^ inflammatory monocytes and interstitial macrophages are the major targets of vaccinia infection in the lungs

**Fig. 6:**
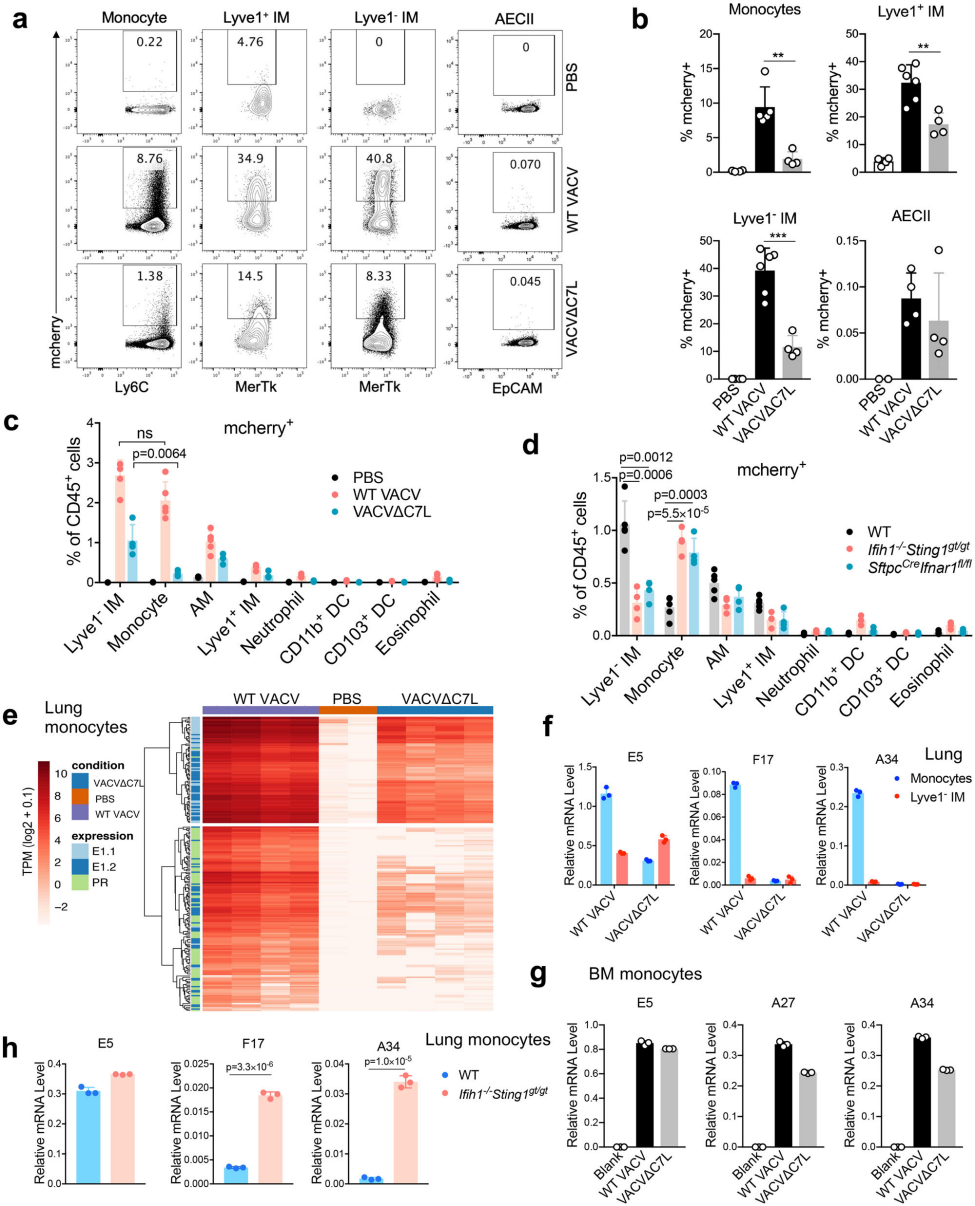
CCR2^+^ inflammatory monocytes and Lyve1^−^ IMs are infected and restrict vaccinia virus life cycle. **a**, **b** Representative flow cytometry dot plots **a** and bar graph **b** showing mCherry^+^ cell populations in the lungs of C57BL/6J mice at day 3 post infection with WT VACV-mCherry, VACV∆C7L-mCherry at 2 × 10^7^ pfu or PBS-mock infected mice (*n* = 4–5 in each group). **c** Bar graph showing mCherry^+^ cells in the lungs of C57BL/6J mice at day 3 post infection with WT VACV-mCherry, VACV∆C7L-mCherry at 2 × 10^7^ pfu or PBS-mock infected mice. **d** Bar graph showing mCherry^+^ cells in the lungs of WT C57BL/6J, *Ifih1*^*−/−*^*Sting1*^*gt/gt*^ or *Sftpc*^*cre*^*Ifnar1*^*fl/fl*^ mice at day 3 post infection with VACV∆C7L-mCherry at 2 × 10^7^ pfu (*n* = 4–5 in each group). **e** Heatmap showing expression levels of viral genes in monocytes isolated from the lung of C57BL/6J mice at day 3 post infection with WT VACV or VACV∆C7L at 2 × 10^7^ pfu. Early gene subclusters were designated E1.1 and E1.2. Intermediate and Late genes were designated as PR: post-replicative. Subsets are derived from^65^. **f** E5, F17 and A34 mRNA expression in monocytes and interstitial macrophages isolated from the lungs of C57BL/6J mice at day 3 post infection with WT VACV or VACV∆C7L at 2 × 10^7^ pfu. **g** E5, F17 and A34 mRNA expression in cultured monocytes infected with either WT VACV or VACV∆C7L at a MOI of 5 for 12 h. **h** E5, A34 and A27 mRNA expression in monocytes and interstitial macrophages isolated from the lungs of C57BL/6J or *Ifih1*^*−/−*^*Sting1*^*gt/gt*^ mice at day 3 post infection with VACV∆C7L at 2 × 10^7^ pfu. **p < 0.01 and ***p < 0.001. Two-tailed unpaired Student’s t test was used for comparisons of two groups in the studies. Data are representative of two **d** or three **a**–**c**, **e**–**h** independent experiments and presented as mean ± SD. Source data are provided as a Source Data file.

To evaluate which cell populations are infected after vaccinia infection, we collected lungs at day three post infection with either WT VACV- or VACV∆C7L-expressing mCherry under the vaccinia synthetic early/late promoter, which drives mCherry expression during early and late viral life cycle. We found that both VACV∆C7L and WT VACV predominantly infected monocytes and IMs after 3 days infection, although WT VACV displayed higher infection efficiency than VACV∆C7L (Fig. 6a, b and Supplementary Fig. 6a, b). The infection rates in lungs AECIIs were very low for both VACV∆C7L and WT VACV (Fig. 6a, b). Whereas similar percentages of Lyve1^−^ IM and monocytes out of CD45^+^ cells were infected with WT VACV, higher percentages of Lyve1^−^ IMs than monocytes out of CD45^+^ cells were infected with VACV∆C7L (Fig. 6c and Supplementary Fig. 6d). VACV∆C7L infection in *Ifih1*^*−/−*^*Sting1*^*gt/gt*^ or *Sftpc*^*cre*^*Ifnar1*^*fl/fl*^ mice showed reduced percentages of mCherry^+^ Lyve1^−^ IMs and increased percentages of mCherry^+^ monocytes compared with those in infected WT mice (Fig. 6d and Supplementary Fig. 6e). These results indicate that monocytes and Lyve1^−^ IMs are the major targets of viral infection in the lungs. Alternatively, these cell populations are more efficient in engulfing infectious viral particles in the lungs.

### CCR2^+^Ly6C^+^ inflammatory monocytes and interstitial macrophages restrict viral life cycle during VACV∆C7L infection

After viral entry, vaccinia virus cores are released into cytoplasm and early gene transcription is initiated after uncoating. After viral DNA replication, post-replicative intermediate and late genes are expressed^32^. To gain insights into the mechanism of monocyte-mediated restriction of vaccinia in vivo, we isolated monocytes from WT VACV or VACV∆C7L-infected lungs at day three post infection. RNA-seq analyses of viral transcriptomes indicated that whereas monocytes isolated from WT VACV-infected lungs expressed early and post-replicative viral intermediate and late genes, those isolated from VACV∆C7L-infected lungs expressed viral early genes but largely failed to express viral intermediate and late genes (Fig. 6e). Our results indicate that monocytes from VACV∆C7L-infected lungs but not those from WT VACV∆C7L-infected lungs restrict viral life cycle.

RT-PCR analyses for selected viral genes validated those findings. Whereas viral early gene E5R expression was detected in monocytes from WT VACV or VACV∆C7L-infected lungs, viral late genes such as F17 and A34 were expressed in monocytes from WT VACV-infected lungs but were not expressed in those from VACV∆C7L-infected lungs (Fig. 6f). By contrast, viral late genes A27 and A34 were expressed in VACV∆C7L-infected monocytes isolated from bone marrow of uninfected mice, indicating the immune environment in the VACV∆C7L-infected lungs endowed the monocytes the ability to restrict viral late gene expression (Fig. 6g). To our surprise, Lyve1^−^ IMs from WT VACV-infected lungs showed stronger ability to restrict viral late gene expression compared with monocytes, as F17 and A34 were not expressed in WT VACV-infected Lyve1^−^ IMs (Fig. 6f). Interestingly, monocytes from VACV∆C7L-infected lungs from *Ifih1*^*−/−*^*Sting1*^*gt/gt*^ mice failed to repress late gene expression (Fig. 6h). Consistent with that, lung monocytes isolated from mice at day 3 post-infection contained more infectious virions compared with Lyve1^−^ IMs, suggesting that Lyve1^−^ IMs are more efficient in eliminating either WT VACV or VACV∆C7L than monocytes (Supplementary Fig. 6g). No infectious virions were detected in lung monocytes or Lyve1^−^ IMs isolated from mice at day 5 post-infection with VACV∆C7L. By contrast, WT VACV persisted in lung monocytes but not in Lyve1^−^ IMs from mice at day 5 post-infection with WT VACV (Supplementary Fig. 6g). In VACV∆C7L-infected *Ifih1*^*−/−*^*Sting1*^*gt/gt*^ mice, we observed infectious virions persisted in monocytes from *Ifih1*^*−/−*^*Sting1*^*gt/gt*^ mice at day 5 post-infection with VACV∆C7L but were cleared in Lyve1^−^ IMs despite of lacking MDA5 and STING (Supplementary Fig. 6h). Taken together, these results provide evidence that monocytes and Lyve1^−^ IMs from VACV∆C7L-infected lungs are endowed with abilities to restrict viral late gene expression and eliminate virions. In addition, nucleic acid-sensing pathways mediated by MDA5 and STING are important for lung inflammatory monocytes to restrict viral life cycle. Above results further confirmed innate immunity mediated by lung AECs in response to VACV∆C7L infection is important for CCR2^+^ monocytes activation and their differentiation into Lyve1^−^ IMs, which are crucial to restrict vaccinia virus infection in the lungs.

## Discussion

Type I IFN and IFN signaling play critical roles in host defense against viral infection^33^. It is not clear what cell types and what pattern-recognition receptors (PRRs) are responsible for IFN production and how IFN exerts its function during a pulmonary poxvirus infection. In this study, we established an acute poxvirus infection model with an attenuated but replication-competent mutant VACV (VACV∆C7L) via the deletion of a host range protein encoded by the C7L gene. VACV∆C7L is attenuated by more than 100-fold compared with WT VACV in WT C57BL/6J mice in an intranasal infection model. Our studies showed that C7 interacts with IRF3 and C7 functions to inhibit IFN production via blocking IRF3 phosphorylation.

**Fig. 7:**
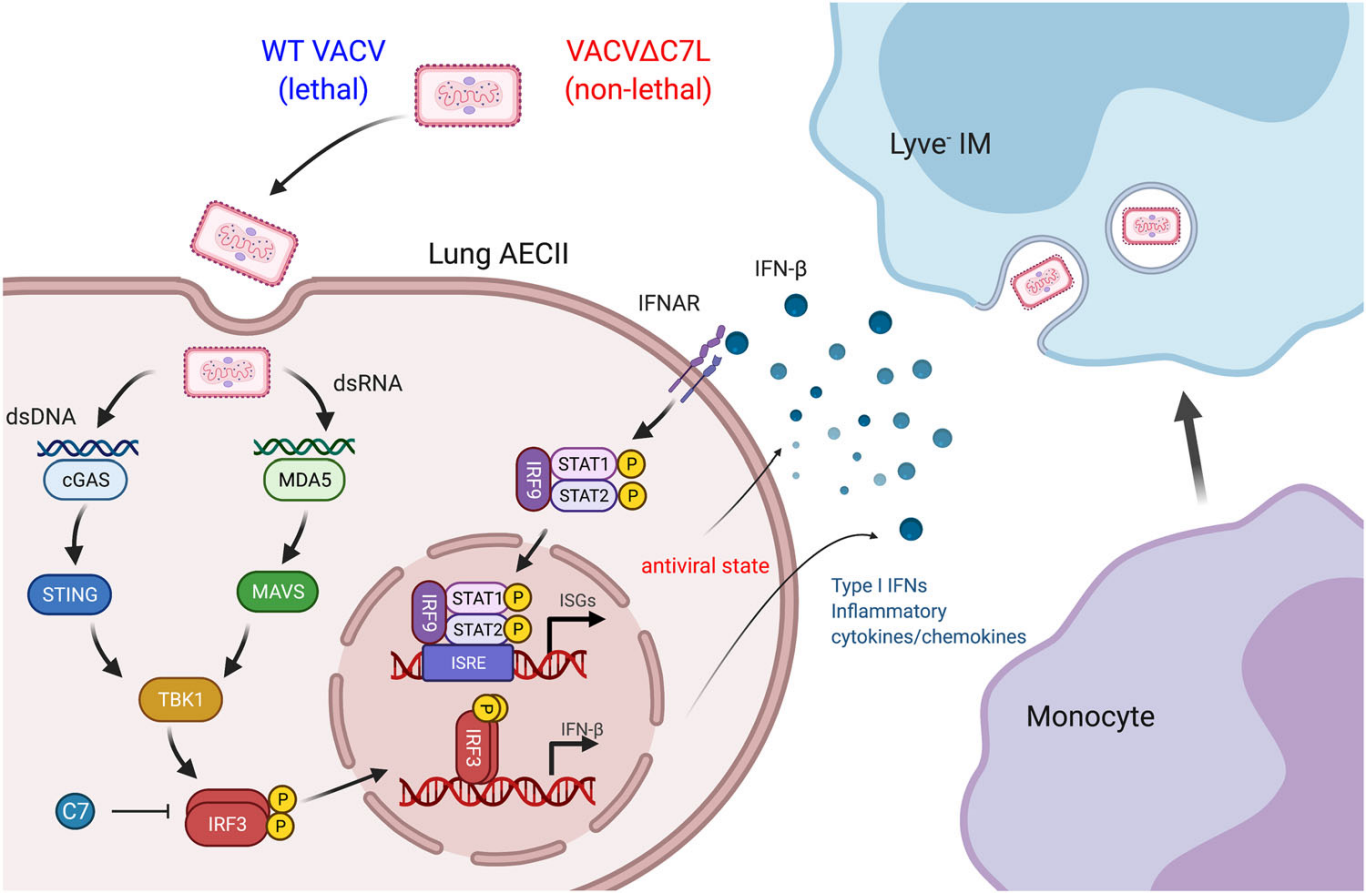
Model of lung innate immune system to protect against vaccinia virus pulmonary infection. Vaccinia C7L encodes a virulence factor. Whereas intranasal infection of WT VACV causes lethality in mice, VACV∆C7L infection is non-lethal. Here we show that C7 blocks the phosphorylation of transcription factor IRF3, which is critical for the induction of IFNB gene expression. VACV∆C7L infects lung AECIIs and triggers IFN-β production via activating the cytosolic DNA and dsRNA-sensing pathways that are dependent on MDA5 and STING. IFN-β-IFNAR signaling on lung AECII further strengthens antiviral effects via the induction of IFN-stimulated genes. Under the influence of various cytokines and chemokines produced by lung AECIIs, CCR2^+^ inflammatory monocytes further differentiate into Lyve1^-^ IMs to fortify host immunity.

We demonstrated that T, B, NK, and alveolar macrophages are dispensable for host defense in this acute viral infection model. In contrast, the innate immune system consisting of lung AECII and CCR2^+^ monocytes have important functions combating acute high-dose infection with this replicative DNA virus. We also demonstrated that both the cytosolic dsRNA-sensing pathway mediated by MDA5 and the cytosolic DNA-sensing pathway mediated by cGAS/STING have important functions in host defense against VACV∆C7L infection. These findings support a model (Fig. 7) in which intranasal infection of VACV∆C7L triggers MDA5/STING-dependent IFN-β production from lung AECII, which strengthens an antiviral state through activating the IFN-β/IFNAR/STAT2 pathway via autocrine and paracrine mechanisms. In the infected lungs and under the influence of various cytokines and chemokines, CCR2^+^ inflammatory monocytes then further differentiate into interstitial macrophages to fortify host immunity by restricting viral replication and dissemination.

Mouse lung epithelial cells and alveolar macrophages provide the front-line defense against invading viral pathogens during pulmonary infection. In the lower respiratory tract, the mouse lung epithelial cell lining is comprised of two main cell types, type I and type II alveolar epithelial cells (AECIs and AECIIs). At steady state, AECIIs have capacity of self-renewing and giving rise to AECIs^34^. AECIIs have been shown to be the major targets of influenza virus infection and contribute to the innate immune defense against viral pathogens both in vitro and in vivo^35–37^. During fungal infection, lung epithelial cells mount robust NF-κB signaling and orchestrate IL-17A and GM-CSF producing innate lymphocytes^38^. The function of lung AECIIs in host defense against poxvirus infection has not been demonstrated previously.

In this study, we found that lung AECIIs have a central function in restricting vaccinia virus infection. AECIIs directly sense VACV∆C7L infection and produce IFN-β and proinflammatory cytokines and chemokines through MDA5 and cGAS/STING-dependent nucleic acid-sensing pathways. MDA5 is a cytosolic sensor of double-stranded RNA (dsRNA), and it is important for host defense against many RNA viruses, including picornavirus, orthomyxovirus, flavivirus, paramyxovirus, and coronavirus families^39–41^. Human MDA5 deficiency has been reported in children with increase susceptibility to viral infections^42–44^. MDA5, but not RIG-I, is involved in detecting modified vaccinia virus Ankara (MVA), a highly attenuated non-replicative vaccinia, infection in macrophages^45^. cGAS/STING pathway in hematopoietic cells is required for host defense against mousepox infection via lympho-hematogenous spread^46,47^. *cGas*^*−/−*^ mice are more susceptible to vaccinia intranasal infection than WT mice^48^. How the cytosolic RNA and DNA-sensing pathways synergize in defending against vaccinia virus infection requires further investigation.

Our results indicate that the type I IFN feedback loop in the non-hematopoietic cell population(s) is most important for mouse survival after VACV∆C7L intranasal infection, although IFNAR1 signaling in hematopoietic cell population(s) also contributes to host defense. Further, we found that AECIIs express high levels of cell surface IFNAR1, and that type I IFN signaling on lung AECIIs is critical for host defense against VACV∆C7L intranasal infection. IFN-β produced by AECIIs may directly stimulate IFNAR1 on AECIIs to produce ISGs that restrict viral replication. Moreover, we found the IFN-β-IFNAR1 signaling on AECIIs is involved in monocytes differentiation into IMs and regulate antiviral function of immune cells in infected tissue.

Monocytes egress from the bone marrow to the blood circulation in response to infection and they also migrate to the infected or inflamed tissue in a CCR2-dependent manner where they further differentiate into other cell types, including inflammatory DCs and macrophages^49,50^. Two functional subsets of mouse blood monocytes were identified: CX_3_CR1^lo^CCR2^+^Gr1^+^ and CX_3_CR1^hi^CCR2^−^Gr1^−^ ^51^. CCR2^+^ monocytes have been shown to be important for antiviral immunity in a mouse model of intravaginal infection with herpes simplex virus 2 and inflammatory monocytes are involved in vaccinia virus intradermal infection^52,53^. In contrast, dysregulated inflammatory monocytes and macrophages may contribute to SARS-CoV or SARS-CoV-2-induced lung immunopathology and death^54–56^. During vaccinia virus infection, we found that CCR2^+^ inflammatory monocytes are indispensable for protection against VACV∆C7L infection. Upon vaccinia virus infection, CCR2^+^ monocytes are recruited into the infected lungs, some of which are differentiated into Lyve1^−^ IMs. CCR2^+^Ly6C^+^ monocytes and differentiated Lyve1^−^ IMs engulf vaccinia virus particles and restrict viral dissemination. The antiviral function of CCR2^+^ monocytes is likely dependent on IFN-β signaling in AECIIs. We speculate that CCL2 and CCL7 produced by lung AECIIs upon VACV∆C7L infection are likely to be involved in the recruitment of CCR2^+^Ly6C^+^ monocytes into the infected areas in the lungs. Further work is needed to elucidate the function of immune cytokines and chemokines secreted by AECIIs contribute to the recruitment and differentiation of inflammatory monocytes.

Our study shows the important function of Lyve1^−^ IMs in restricting vaccinia infection in the lungs. Using *Ccr2-GFP* mice, we showed that upon intranasal VACV∆C7L infection, the CCR2^+^ monocytes can be differentiated into Lyve1^−^ IMs. IFN production and signaling by lung AECII are important for the differentiation of CCR2^+^ monocytes into Lyve1^−^ IMs. In addition, Lyve1^−^ IMs are major targets of vaccinia infection in the lungs and they play important antiviral function by restricting viral life cycle. Recent studies showed that IMs are complex cell populations and exhibit differences in tissue locations, phenotypes, transcriptomes, and functions^30,31,57–60^. It is still unclear how Lyve1^−^ IMs perform their anti-vaccinia virus function and  how IFN-β production and signaling in AECIIs promotes CCR2^+^ monocytes differentiation into Lyve1^−^ IMs.

Taken together, our results highlight the importance of type I IFN production and IFNAR signaling in the lung AECIIs during the host defense against pulmonary vaccinia infection. It is crucial to understand why type I IFN is important for host restriction of vaccinia infection, and yet detrimental in the case of SARS-CoV and SARS-CoV-2 infection^54,55^. Like SARS-CoV, SARS-CoV-2 replicates rapidly in the infected lungs and suppresses IFN induction. This delayed induction of IFN leads to recruitment and activation of inflammatory monocytes and macrophages in the infected lungs resulting in lung damage^54,61^. Whether monocytes and IMs have a crucial function protecting against SARS-CoV-2 is still unknown.

Although the current study is limited to vaccinia virus pathogenesis, given the importance of nucleic acid-sensing pathways and type I IFN signaling in antiviral immunity, we suspect that the cross-talk between AECII and inflammatory CCR2^+^ monocytes would be important for host defense against other pulmonary viral infections. More efforts will be needed to explore the differences in the transcriptomes and functionalities of monocytes and IMs after vaccinia virus infection to gain a deeper understanding of how those cells restrict virus infection and clear viral particles in the lungs. scRNA-seq analyses will be performed in future studies to obtain more comprehensive knowledge about what cell types are infected by the virus, what transcriptomic changes occur in lung resident and immune cells after virus infection, and how IFN induction and signaling affects the transcriptomic landscape.

In summary, we identified vaccinia C7 as a critical virulence factor for lethal infection. Using an attenuated vaccinia virus (VACV∆C7L), we established an intranasal poxvirus virus infection model to focus on the function of host innate immunity in restricting viral infection in the lung. Our results highlight the cross-talk between lung AECs with CCR2^+^ monocytes in the control of acute pulmonary poxvirus infection. These findings have broad implications in understanding pathophysiology of viral pneumonia and for developing effective therapeutic strategies.

## Methods

### Mice

Female C57BL/6J mice between 6 and 8 weeks of age were purchased from the Jackson Laboratory and were used for the preparation of bone marrow-derived dendritic cells and for intranasal infection experiments. *Ifnb1*^*Eyfp*^ reporter mice, *cGas*^*−/−*^, *Stat2*^*−/−*^, *Ifnar1*^*−/−*^, *Sftpc-CreER*^*T2*^, *Ifnar*^*fl/fl*^, *Scgb1a1-CreER*^*T*M^, *Rosa26-lox-stop-lox-TdTomato* mice were purchased from the Jackson Laboratory. *Sting1*^*gt/gt*^ mice^62^ were generated in the laboratory of Russell Vance (University of California, Berkeley). *Ifih1*^*−/−*^ (MDA5 knockout) mice^40^ were generated in Marco Colonna’s laboratory (Washington University). *Ifih1*^*−/−*^*Sting1*^*gt/gt*^, *Scgb1a1*^*Cre*^*R26*^*tdT*^ and *Sftpc*^*cre*^*Ifnar1*^*fl/fl*^ mice were bred in our lab. *Ccr2-GFP* and *Ccr2-DTR* mice^29^ were provided by Eric Pamer (University of Chicago). *Irf3*^*−/−*^ mice were provided by Ruslan Medzhitov (Yale University). All transgenic mice are from C57BJ/6J background. These mice were maintained in the animal facility at the Sloan Kettering Cancer Institute. All procedures were performed in strict accordance with the recommendations in the Guide for the Care and Use of Laboratory Animals of the National Institute of Health. The protocol was approved by the Committee on the Ethics of Animal Experiments of Sloan-Kettering Cancer Institute.

### Viruses

The Western Reserve (WR) strain of vaccinia virus (VACV) was propagated and virus titers were determined on BSC40 (African green monkey kidney cells) monolayers at 37 °C. MVA virus was kindly provided by Gerd Sutter (University of Munich), and propagated in BHK-21 (baby hamster kidney cell, ATCC CCL-10) cells. The viruses were purified through a 36% sucrose cushion. Heat-iMVA was generated by incubating purified MVA virus at 55 °C for 1 h. Sendai virus (SeV; Cantell strain) was purchased from Charles River Laboratories. To generate recombinant VAC∆C7L virus, BSC40 cells were infected with WT vaccinia virus WR strain at a MOI of 0.2. After 1–2 h, cells were transfected with pC7-GFP or pC7-mCherry plasmids with lipofectamine 2000. Homologous recombination between the plasmid DNA and viral genome results in the deletion of C7L gene from the viral genome and insertion of either GFP or mCherry which is under the control of vaccinia synthetic early and late promoter (pSE/L). Cells were collected two days later and underwent three cycles of freeze-thaw. Plaque purification was performed based on the GFP or mCherry expression under the microscope. After 4-5 rounds, pure recombinant VACV∆C7L-GFP and VACV∆C7L-mCherry were obtained. PCR analyses and DNA sequencing were performed to make sure that the C7L gene was deleted from the VACV genome. MVA∆C7L expressing GFP was generated in BHK21 cells following similar procedure as described above.

### Intranasal infection of WT VACV or VACV∆C7L in mice

C57BL/6J mice between 6 and 8 weeks of age (5–10 in each group) were anesthetized and infected intranasally with increasing doses of WT VACV or VACV∆C7L at indicated pfu in 20 µl PBS (with 10 µl to each nostril). In most of the experiments, female mice were used. In one experiment, female and male mice were used to compare their susceptibility to VACV∆C7L infection. Mice were monitored and weight daily. Mice that had lost over 30% of initial weight were euthanized. Kaplan-Meier survival curves were determined. To measure viral titers within different organs, lungs, livers, brains and spleens were harvested, placed into tubes with 1 ml of PBS, and homogenized using the Miltenyi GentleMACS Dissociator. Cell suspensions underwent freeze-thaw cycles three times and sonicated before titration on BSC40 cells. Blood was collected into 1.5 ml eppendorf tubes and serum were kept after centrifugation. Virus titers were determined by calculating the number of plaques (pfu) per gram of tissues (pfu/g).

### Cell lines and primary cells

BSC40, HEK293T, and RAW264.7 were cultured in Dulbecco’s modified Eagle’s medium supplemented with 10% fetal bovine serum (FBS), 2 mM L-glutamine and 1% penicillin-streptomycin. BHK-21 were cultured in Eagle’s Minimal Essential Medium (Eagle’s MEM, Life Technologies, Cat# 11095-080) containing 10% FBS, with 100 U/ml penicillin and 100 μg/ml streptomycin. THP-1 cells were treated with PMA (10 ng/ml) for 72 h for differentiation into macrophages. For the generation of GM-CSF-BMDCs, the bone marrow cells (5 million cells in each 15 cm cell culture dish) were cultured in RPMI-1640 medium supplemented with 10% fetal bovine serum (FBS) in the presence of GM-CSF (30 ng/ml, produced by the Monoclonal Antibody Core facility at the Sloan Kettering Institute) for 9–12 days. Primary mouse monocytes were isolated from C57BL/6J bone marrow using the Monocyte Isolation Kit (Miltenyi Biotec, 130-100-629) and replated in RPMI with 10% FBS.

To culture primary mouse AEC2, the lungs from euthanized female C57BL/6J mice between 6 and 8 weeks of age were perfused via the right ventricle with 10 ml PBS, then inflated with a 1.5 ml mixture of 0.5 ml low melting agarose (1% w/v) and 1 ml dispase (Corning, 25 U/ml). The lung lobes were gently minced into small pieces in a conical tube containing 3 ml of PBS, 25 U/mL of dispase (Roche), and 100 U/ml DNase I (Sigma) followed by incubation on a rotator at 37 °C for 45 min. The cells were filtered through 40 μm mesh and for further staining against antibodies for flow cytometry: pan CD45-APC, CD31-APC, FITC-CD104 and EpCAM-APC-Cy7 (BioLegend). LIVE/DEAD Fixable Aqua Stain (ThermoFisher) was used to eliminate dead cells. Cell sorting was performed with a FACS Aria II (BD Biosciences). AECII progenitors cells were plated into a TC plate pre-coated with Matrigel (Corning, 354230) and cultured with Small Airway Epithelial Cell Growth Medium (Lonza) supplemented with charcoal-stripped 5% FBS, 10 ng/ml keratinocyte growth factor (PeproTech, 100-19), 10 μM Rock inhibitor (Selleck Chemicals, S1049), with 100 U/ml penicillin and 100 μg/ml streptomycin at 37 °C in a 5% CO_2_ incubator for the first 2 days, and then replaced with the same media but without Rock inhibitor for the next 4 to 5 days.

### Multistep growth curve of WT VACV and VACV∆C7L

BSC40 or lung AECII cells were infected with WT VACV or VACV∆C7L at a MOI of 0.05. The cells were then scraped into the medium and collected at indicated times. After three cycles of freeze-thaw and subsequent sonication, viral titers in the collected samples were determined by plaque assay on BSC40 cells.

### Cytokine assays

The cytokine levels in bronchoalveolar lavage fluids were determined by ELISA for IFN-α and IFN-β (PBL Biomedical Laboratories) and by Cytokine Mouse Magnetic 20-Plex Panel (ThermoFisher). To determine the cytokine levels in the supernatants of cultured cells, lung AECII, bone marrow monocytes, and bone marrow-derived dendritic cells were infected with either WT VACV or VACV∆C7L at a MOI of 5. Supernatants were collected at 24 h post infection. The levels of IFN-β, CCL4, and CCL5 were determined by ELISA (R&D for CCL4 and CCL5).

### Flow cytometry

To prepare single cell suspension from the BALF and lungs, bronchoalveolar lavage fluid (BALF) was harvested with intratracheal infusion of 1 ml of cold PBS. To harvest lungs, lungs were cleared of blood with perfusion of cold PBS through the right ventricle. The lung lobes were excised and digested with Collagenase D (2 mg/ml) and DNase I (100 μg/ml) for 45 min at 37 °C. After dissociation of the lung tissue in the Miltenyi GentleMACS Dissociator, lung homogenates were incubated with red blood cell lysis buffer on ice for 5 min and then quenched with cold PBS. The cell pellets were resuspended with MACS buffer (Miltenyi Biotec) to generate single cell suspension and then filtered through 70 μm nylon mesh prior to FACS analysis.

To analyze cell populations in BALF and lungs, single cell suspensions were blocked by using anti-CD16/CD32 antibody and stained with various antibodies on ice for 30 mins. LIVE/DEAD Fixable Aqua Stain (ThermoFisher) was used to stain dead cells. For intracellular cytokine staining, lung cell suspensions were incubated with 5 μg/ml peptide (B8R 20-27 or OVA 257-264) and Brefeldin A (0.1%) at 37 °C for 6 h prior to all staining, then treated with BD Cytofix/Cytoperm kit for staining. The antigens and clone designations for the antibodies were as follows: BioLegend: CD45.2 (104), CD45 (30-F11), CD45.1 (A20), EpCAM (G8.8), CD31 (MEC13.3), CD104 (346-11A), T1a (8.1.1), Ly6G (1A8), CD11c (N418), CD11b (M1/70), MHC II (M5/114.15.2), CD64 (X54-5/7.1), CD3e (145-2C11), CD4 (GK1.5), CD8 (53-5.8) IFN-γ (XMG1.2), IFNAR-1 (MAR1-5A3), BD Biosciences: CD45 (30-F11), Siglec F (E50-2440), CD19 (1D3), CD49b (DX5), Thermo Fisher: CD16/CD32 (93), Ly6C (HK1.4), MerTk (DS5MMER), Lyve1 (ALY7). All antibodies were used at 1:200. Cells were analyzed on the BD LSRFortessa or LSR II flow cytometer. Cell sorting was performed with a FACS Aria II (BD Biosciences) and data were analyzed with FlowJo software (version 10.5.3).

### Immunofluorescence

Lungs were perfused and fixed with 4% paraformaldehyde for overnight at 4 °C. For *Ifnb1*^*Eyfp*^ reporter mice, after embedded in O.C.T, cryosections (10 μm) were used for immunofluorescent (IF) analysis. For *Ccr2-GFP* mice, paraffin sections (6 μm) were used. Cultured cells were plated in Lab-Tek Π chamber (ThermoFisher) and fixed with 4% PFA at RT for 10 min. Tissue sections or cells were permeabilized with 0.5% Triton X-100 in PBS for 5 min and then blocked in 5% goat serum (Sigma), 3% bovine serum albumin (Fisher) and 0.1% Triton X-100 at room temperature for 1 hr. Primary antibodies were incubated at 4 °C at the indicated dilutions overnight: chicken anti-GFP (1:1000, Abcam), rabbit anti-SP-C (1:1000, Millipore), rabbit anti-C7 (1:500), mouse anti-dsRNA (1:100, Millipore). Alexa Fluor-coupled secondary antibodies (1:1000, Invitrogen) were incubated at room temperature for 60 min. After antibody staining, sections were embedded in ProLong Gold Antifade Mountant (ThermoFisher). Images were acquired using a confocal microscope (Leica TCS SP8). All the images were further processed with Image J software.

### Tamoxifen, diphtheria toxin administration and immune cells depletion

Diphtheria toxin (DT) was obtained from Sigma, reconstituted at 1 mg/ml in PBS, and frozen in aliquotes at −80 °C. Mice received 10 ng/g DT intraperitoneally in 100 µl of PBS at day −1, 1, and 3 relative to virus inoculation. Tamoxifen (Sigma) was made in 40 mg/ml stock solution in corn oil (Sigma) and was given 4 mg per mouse via intraperitoneal injection ×4 doses. For NK depletion experiments, mice were injected with 200 μg/mouse of anti-NK1.1 (clone PK136, BioXcell) or similar amounts of appropriate control antibody per injection at day −2, 0. To deplete AM, 50 μl clodronate-containing liposomes (FormuMax) in endotoxin-free PBS was administered intranasally to anesthetized mice at day −2, 0.

### Bone marrow chimeric mice

Wild-type B6.SJL mice (*CD45.1* background) or *Ifnar1*^*−/−*^ mice (*CD45.2* background) were given a dose of irradiation (1096 Rads). After 6 h, mice were injected retro-orbitally with isolated WT or KO bone marrow cells (5 × 10^6^ cells per mouse). Antibiotic-containing water (80 mg/L trimethoprim and 400 mg/L sulfamethoxazole) were provided daily for four weeks after irradiation. After another 4 weeks, the mice are ready for experiments.

### Plasmid construction

IFN-β reporter plasmid (pIFN-β-luc)^63^ and NF-κB reporter plasmid were provided by Michaela Gack (University of Chicago). STING, TBK1, IRF3^64^ were provided by Tom Maniatis (University of Columbia). IRF3-5D^26^ were provided by Rongtuan Lin (McGill University). MAVS, TLR3, TRIF plasmids were purchased from Addgene. VACV C7L was amplified by PCR from VACV WR genome and subcloned into pcDNA3.1 and pQCXIP.

### Dual Luciferase reporter assay

Luciferase activities were measured using the Dual Luciferase Reporter Assay system according to the manufacturer’s instructions (Promega). Briefly, expression plasmids including a firefly luciferase reporter construct, a *Renilla* luciferase reporter construct, as well as other expression constructs were transfected into HEK293T cells. 24 h post transfection, cells were collected and lysed. The relative luciferase activity was expressed as arbitrary units by normalizing firefly luciferase activity under *Ifnb1* promoter to Renilla luciferase activity from a control plasmid pRL-TK.

### Construction of retrovirus expressing vaccinia C7L

HEK293T cells were passaged into 6-well plate. Next day, cells were transfected with three plasmids- VSVG, gag/pol and pQCXIP-C7 or pQCXIP with lipofectamine 2000. After 2 days, cell supernatants were collected and filtered through 0.45 μm filter and stored in −80 °C.

### RAW264.7 and THP-1 cell line stably expressing vaccinia C7L

**C**ells were passaged into 6-well plate. Next day, cells were infected with retrovirus expressing C7L or control virus at MOI 5. After 2 days, culture medium was replaced with medium containing puromycin. After one week, survival cells are the cells stably expressing C7L and verified by Western blot analysis using anti-C7 antibody.

### Generation of VACV C7 specific polyclonal antibodies

The vaccinia C7L gene was cloned into bacterial expression vector-pET28-SUMO. The C7 expression plasmids were transfected into *E. coli* BL21 (DE3) cells. Cultures (2-liter) from a single transformant were grown at 37 °C in LB Broth containing 100 μg/ml ampicillin until the *A*_600_ reached 0.6. The cultures were adjusted to 0.5 mM isopropyl-β-d-thiogalactopyranoside (IPTG), and then incubated for 20 h at 18 °C with constant shaking. Cells were harvested by centrifugation and resuspended in buffer A (50 mM Tris–HCl, pH 7.5, 500 mM NaCl, 20 mM imidazole, 10% glycerol). The cells were lysed by sonication and the insoluble material was removed by centrifugation at 12,000 g for 45 min. The bacterial lysates were mixed for 1 h with 5 ml of Ni-NTA resin (Qiagen) that had been equilibrated with buffer A. The resins were poured into gravity-flow columns and then washed with 60 ml of buffer A. The adsorbed proteins were step-eluted with 300 mM imidazole in buffer A. The polypeptide compositions of the eluate fractions were monitored by SDS-PAGE and the peak fractions containing each recombinant protein were pooled. The eluates were dialyzed against buffer containing 50 mM Tris-HCl (pH 8), 200 mM NaCl, 2 mM DTT, 2 mM EDTA, 10% glycerol, and 0.1% Triton X-100 and then stored at –80 °C. Rabbit immunization for the generation of anti-C7 polyclonal rabbit antibody was performed in Pocono Rabbit Farm and Laboratory (PRF&L). The anti-C7 antibodies were purified from rabbit serum using affinity purification.

### Western blot analysis

Cells were lysed in RIPA lysis buffer supplemented with 1x Halt Protease and Phosphatase Inhibitor Cocktail. Protein samples were separated by SDS-PAGE and then transferred to nitrocellulose membrane. Primary antibodies specific for phospho-IRF3 (1:1000, CST, 4947), IRF3 (1:1000, CST, 4302), IRF3 (1:1000, Millipore, MABF268), C7 antibody (1:500), FLAG (1:1000, Sigma, F3165), GAPDH (1:2000, CST, 2118) and β-actin (1:2000, CST, 4967) were use. β-actin or GAPDH were used as loading controls. Anti-rabbit or mouse HRP-linked IgG antibody was used as a secondary antibody (1:5000, CST, 7074 or 7076). Detection was performed using SuperSignal Substrates (Thermo Fisher, 34577 or 34095).

### Co-immunoprecipitation

HEK293T cells in 10 cm plates were transfected with Flag-IRF3 together with pcDNA3.1-C7L. Two days later, cells were lysed in Pierce IP lysis buffer on ice for 30 min. For AECII cells, they were infected with WT VACV or VACV∆C7L at a MOI of 5 for 6 h. Cells were lysed as above. C7 antibody was added into cell lysate to a final concentration of 1 μg/ml and incubated at 4 °C overnight on a rotator. Next day, protein A-Magnetic beads was added and incubated at 4 °C for 2 h. The beads were washed five times with IP lysis buffer. Lastly, the proteins in SDS buffer were denatured by heating at 98 °C for 5 min before they were loaded on a SDS-PAGE.

### Quantitative real-time PCR

Total RNA was obtained from cultured cells with TRIzol reagent (Invitrogen) and extracted using RNeasy Micro Kit (Qiagen) or RNeasy Mini Kit (Qiagen). Cellular RNAs were reverse-transcribed and amplified by PCR using the Verso cDNA synthesis kit (Thermo Fisher) and SYBR Green Master Mix (Thermo Fisher). Cellular RNAs were normalized to GAPDH levels. All assays were performed on an ABI 7500 system and analyzed with ABI 7500 SDS software v.1.3. The primers used are listed in Table S1.

### RNA sequencing and analysis

Total RNA was extracted from collected cells at indicated time points using RNeasy Micro Kit (Qiagen) according to manufacturer’s protocol including DNase I treatment. All RNAs were analyzed on an Agilent Bioanalyser for quality assessment. Libraries were constructed using TruSeq RNA Library Prep Kits (Illumina) or NEBNext Ultra II Directional RNA Library Prep Kits (NEB) poly-A mRNA selection modules respective to each kit. Finished pooled libraries were sequenced at the Rockefeller University Genomics Resource Center on a NextSeq500 instrument (Illumina).

The resulting FASTQ files were processed using kallisto (PMID: 27043002) followed by a tximport (PMID: 26925227) transcript-to-gene level transformation. Transcript indices for kallisto were created using FASTA files from Ensembl release 95 for mm10 for all annotated cDNA and ncRNA transcripts, as well as FASTA sequences for WT VACV transcripts (accession no. NC_006998.1). Gene-level read counts were then processed using the limma suite of tools (PMID: 25605792) first with a voom transformation, followed by linear model fitting to determine differentially expressed genes, and lastly performing gene set testing using the CAMERA function. Select gene sets were plotted as row normalized z-scores in heatmaps using voom normalized counts. Volcano plots were generated using ggplot2 in R using corrected *p*-values and expression fold changes after linear model fitting for the relevant comparison. Select genes corresponding to most enriched or de-enriched gene sets highlighted. For viral transcriptome analyses, viral gene sets were derived form (PMC2895082) and plotted as transcript per million (tpm) mapped reads.

### Statistical analysis

Two-tailed unpaired Student’s t test was used for comparisons of two groups in the studies. Survival data were analyzed by log-rank (Mantel-Cox) test. The *p* values deemed significant are indicated in the figures as follows: *p < 0.05; **p < 0.01; ***p < 0.001; ****p < 0.0001. Statistical analyses were performed on the Prism 7.0a software (GraphPad Software). Detailed information of the statistical test, the numbers of animals, biological replicates, technical replicates, the number of independent experiments included in the study are discussed in each figure legend.

### Biological materials

All unique materials are readily available from the corresponding authors on request. The availability of the antibody recognizing VACV protein C7 is limited.

### Reporting summary

Further information on research design is available in the Nature Research Reporting Summary linked to this article.

## Supplementary information

Supplementary Information

Reporting Summary

## Data Availability

RNAseq data have been deposited in the Gene Expression Omnibus (GEO) under the accession number GSE158267. Poxvirus nucleotide sequences mentioned in this study are publicly available on NCBI GenBank: VACV Western Reserve (NC_006998.1). All the other data supporting the findings of this study is available within the article and its supplementary information files. Source data are provided with this paper.

## Source data

Source Data

## References

[1] Iwasaki A, Medzhitov R (2015). Control of adaptive immunity by the innate immune system. Nat. Immunol..

[2] Fenner, F. *Smallpox and its eradication*. World Health Organization: Geneva, 1988.

[3] Bray M, Buller M (2004). Looking back at smallpox. Clin. Infect. Dis..

[4] Monkeypox. *World Health Organization* (2021).

[5] Simpson K (2020). Human monkeypox - After 40 years, an unintended consequence of smallpox eradication. Vaccine.

[6] Turner GS (1967). Respiratory infection of mice with vaccinia virus. J. Gen. Virol..

[7] Hayasaka D, Ennis FA, Terajima M (2007). Pathogeneses of respiratory infections with virulent and attenuated vaccinia viruses. Virol. J..

[8] Fenner F (1958). The biological characters of several strains of vaccinia, cowpox and rabbitpox viruses. Virology.

[9] Goritzka M (2015). Alveolar macrophage-derived type I interferons orchestrate innate immunity to RSV through recruitment of antiviral monocytes. J. Exp. Med..

[10] Stegemann-Koniszewski S (2016). Alveolar type II epithelial cells contribute to the anti-influenza A virus response in the lung by integrating pathogen- and microenvironment-derived signals. mBio.

[11] Muller U (1994). Functional role of type I and type II interferons in antiviral defense. Science.

[12] Garcia-Sastre A (2017). Ten strategies of interferon evasion by viruses. Cell Host Microbe.

[13] Perkus ME (1990). Vaccinia virus host range genes. Virology.

[14] Sivan G, Ormanoglu P, Buehler EC, Martin SE, Moss B (2015). Identification of restriction factors by human genome-wide RNA interference screening of viral host range mutants exemplified by discovery of SAMD9 and WDR6 as inhibitors of the vaccinia virus K1L-C7L- mutant. mBio.

[15] Meng X (2018). A paralogous pair of mammalian host restriction factors form a critical host barrier against poxvirus infection. PLoS Pathog..

[16] Lehmann MH (2016). CCL2 expression is mediated by type I IFN receptor and recruits NK and T cells to the lung during MVA infection. J. Leukoc. Biol..

[17] Schoggins JW (2011). A diverse range of gene products are effectors of the type I interferon antiviral response. Nature.

[18] Liu SY, Sanchez DJ, Aliyari R, Lu S, Cheng G (2012). Systematic identification of type I and type II interferon-induced antiviral factors. Proc. Natl Acad. Sci. USA.

[19] Wu J, Chen ZJ (2014). Innate immune sensing and signaling of cytosolic nucleic acids. Annu Rev. Immunol..

[20] Volz A, Sutter G (2017). Modified vaccinia virus ankara: history, value in basic research, and current perspectives for vaccine development. Adv. Virus Res..

[21] Desai TJ, Brownfield DG, Krasnow MA (2014). Alveolar progenitor and stem cells in lung development, renewal and cancer. Nature.

[22] Fehrenbach H (2001). Alveolar epithelial type II cell: defender of the alveolus revisited. Respir. Res..

[23] Unkel B (2012). Alveolar epithelial cells orchestrate DC function in murine viral pneumonia. J. Clin. Invest..

[24] Vaughan AE (2015). Lineage-negative progenitors mobilize to regenerate lung epithelium after major injury. Nature.

[25] Chapman HA (2011). Integrin alpha6beta4 identifies an adult distal lung epithelial population with regenerative potential in mice. J. Clin. Invest..

[26] Lin R, Heylbroeck C, Pitha PM, Hiscott J (1998). Virus-dependent phosphorylation of the IRF-3 transcription factor regulates nuclear translocation, transactivation potential, and proteasome-mediated degradation. Mol. Cell Biol..

[27] Rock JR (2011). Multiple stromal populations contribute to pulmonary fibrosis without evidence for epithelial to mesenchymal transition. Proc. Natl Acad. Sci. USA.

[28] Tscharke DC (2005). Identification of poxvirus CD8+ T cell determinants to enable rational design and characterization of smallpox vaccines. J. Exp. Med.

[29] Hohl TM (2009). Inflammatory monocytes facilitate adaptive CD4 T cell responses during respiratory fungal infection. Cell host microbe.

[30] Chakarov S (2019). Two distinct interstitial macrophage populations coexist across tissues in specific subtissular niches. Science.

[31] Gibbings SL (2017). Three unique interstitial macrophages in the murine lung at steady state. Am. J. Respir. Cell Mol. Biol..

[32] Moss B (1990). Regulation of vaccinia virus transcription. Annu Rev. Biochem..

[33] Schneider WM, Chevillotte MD, Rice CM (2014). Interferon-stimulated genes: a complex web of host defenses. Annu Rev. Immunol..

[34] Hogan BL (2014). Repair and regeneration of the respiratory system: complexity, plasticity, and mechanisms of lung stem cell function. Cell Stem Cell.

[35] Weinheimer VK (2012). Influenza A viruses target type II pneumocytes in the human lung. J. Infect. Dis..

[36] Yu WC (2011). Viral replication and innate host responses in primary human alveolar epithelial cells and alveolar macrophages infected with influenza H5N1 and H1N1 viruses. J. Virol..

[37] Galani IE (2017). Interferon-lambda mediates non-redundant front-line antiviral protection against influenza virus infection without compromising host fitness. Immunity.

[38] Hernandez-Santos N (2018). Lung epithelial cells coordinate innate lymphocytes and immunity against pulmonary fungal infection. Cell Host Microbe.

[39] Kato H (2006). Differential roles of MDA5 and RIG-I helicases in the recognition of RNA viruses. Nature.

[40] Gitlin L (2006). Essential role of mda-5 in type I IFN responses to polyriboinosinic:polyribocytidylic acid and encephalomyocarditis picornavirus. Proc. Natl Acad. Sci. USA.

[41] Dias Junior AG, Sampaio NG, Rehwinkel J (2019). A balancing act: MDA5 in antiviral immunity and autoinflammation. Trends Microbiol..

[42] Zaki M (2017). Recurrent and prolonged infections in a child with a homozygous IFIH1 nonsense mutation. Front Genet..

[43] Lamborn IT (2017). Recurrent rhinovirus infections in a child with inherited MDA5 deficiency. J. Exp. Med..

[44] Asgari S (2017). Severe viral respiratory infections in children with IFIH1 loss-of-function mutations. Proc. Natl Acad. Sci. USA.

[45] Delaloye J (2009). Innate immune sensing of modified vaccinia virus Ankara (MVA) is mediated by TLR2-TLR6, MDA-5 and the NALP3 inflammasome. PLoS Pathog..

[46] Xu RH (2015). Sequential activation of two pathogen-sensing pathways required for type I interferon expression and resistance to an acute DNA virus infection. Immunity.

[47] Wong EB, Montoya B, Ferez M, Stotesbury C, Sigal LJ (2019). Resistance to ectromelia virus infection requires cGAS in bone marrow-derived cells which can be bypassed with cGAMP therapy. PLoS Pathog..

[48] Schoggins JW (2014). Pan-viral specificity of IFN-induced genes reveals new roles for cGAS in innate immunity. Nature.

[49] Serbina NV, Jia T, Hohl TM, Pamer EG (2008). Monocyte-mediated defense against microbial pathogens. Annu Rev. Immunol..

[50] Shi C, Pamer EG (2011). Monocyte recruitment during infection and inflammation. Nat. Rev. Immunol..

[51] Geissmann F, Jung S, Littman DR (2003). Blood monocytes consist of two principal subsets with distinct migratory properties. Immunity.

[52] Iijima N, Mattei LM, Iwasaki A (2011). Recruited inflammatory monocytes stimulate antiviral Th1 immunity in infected tissue. Proc. Natl Acad. Sci. USA.

[53] Hickman HD (2013). Anatomically restricted synergistic antiviral activities of innate and adaptive immune cells in the skin. Cell Host Microbe.

[54] Channappanavar R (2016). Dysregulated Type I interferon and inflammatory monocyte-macrophage responses cause lethal pneumonia in SARS-CoV-infected mice. Cell Host Microbe.

[55] Israelow B (2020). Mouse model of SARS-CoV-2 reveals inflammatory role of type I interferon signaling. J. Exp. Med..

[56] Blanco-Melo D (2020). Imbalanced host response to SARS-CoV-2 drives development of COVID-19. Cell.

[57] Schyns J (2019). Non-classical tissue monocytes and two functionally distinct populations of interstitial macrophages populate the mouse lung. Nat. Commun..

[58] Ural BB (2020). Identification of a nerve-associated, lung-resident interstitial macrophage subset with distinct localization and immunoregulatory properties. Sci. Immunol..

[59] Sajti E (2020). Transcriptomic and epigenetic mechanisms underlying myeloid diversity in the lung. Nat. Immunol..

[60] Aran D (2019). Reference-based analysis of lung single-cell sequencing reveals a transitional profibrotic macrophage. Nat. Immunol..

[61] Merad M, Martin JC (2020). Pathological inflammation in patients with COVID-19: a key role for monocytes and macrophages. Nat. Rev. Immunol..

[62] Sauer JD (2011). The N-ethyl-N-nitrosourea-induced Goldenticket mouse mutant reveals an essential function of Sting in the in vivo interferon response to Listeria monocytogenes and cyclic dinucleotides. Infect. Immun..

[63] Wies E (2013). Dephosphorylation of the RNA sensors RIG-I and MDA5 by the phosphatase PP1 is essential for innate immune signaling. Immunity.

[64] Fitzgerald KA (2003). IKKepsilon and TBK1 are essential components of the IRF3 signaling pathway. Nat. Immunol..

[65] Yang Z, Bruno DP, Martens CA, Porcella SF, Moss B (2010). Simultaneous high-resolution analysis of vaccinia virus and host cell transcriptomes by deep RNA sequencing. Proc. Natl Acad. Sci. USA.

