# Supplementary material for: Lung type II alveolar epithelial cells collaborate with CCR2+ inflammatory monocytes in host defense against poxvirus infection
Source: Nat Commun. 2022 Mar 29;13:1671. doi: 10.1038/s41467-022-29308-2 (PMC8964745; doi:10.1038/s41467-022-29308-2)
Supplement: Supplementary file 1 — Supplementary Information [file 41467_2022_29308_MOESM1_ESM.pdf]

Supplementary Information

Lung type II alveolar epithelial cells collaborate with CCR2$^+$ inflammatory monocytes in host defense against poxvirus infection

Ning Yang[1*], Joseph M. Luna[2], Peihong Dai[1], Yi Wang[1], Charles M. Rice[2], and Liang Deng[1,2,3*#]

[1]Dermatology Service, Department of Medicine, Memorial Sloan Kettering Cancer Center, New York, New York, USA.

[2]Laboratory of Virology and Infectious Disease, The Rockefeller University, New York, NY, United States.

[3]Weill Cornell Medical College, New York, New York, USA.

*Corresponding authors. #Lead contact. Mailing address for Liang Deng and Ning Yang: Dermatology Service, Department of Medicine, Memorial Sloan Kettering Cancer Center, 1275 York Ave., New York, NY 10065. Email: dengl@mskcc.org; yangn@mskcc.org

This file contains:

- Supplementary Fig. 1
- Supplementary Fig. 2
- Supplementary Fig. 3
- Supplementary Fig. 4
- Supplementary Fig. 5
- Supplementary Fig. 6
- Supplementary Table 1

Supplementary Fig. 1

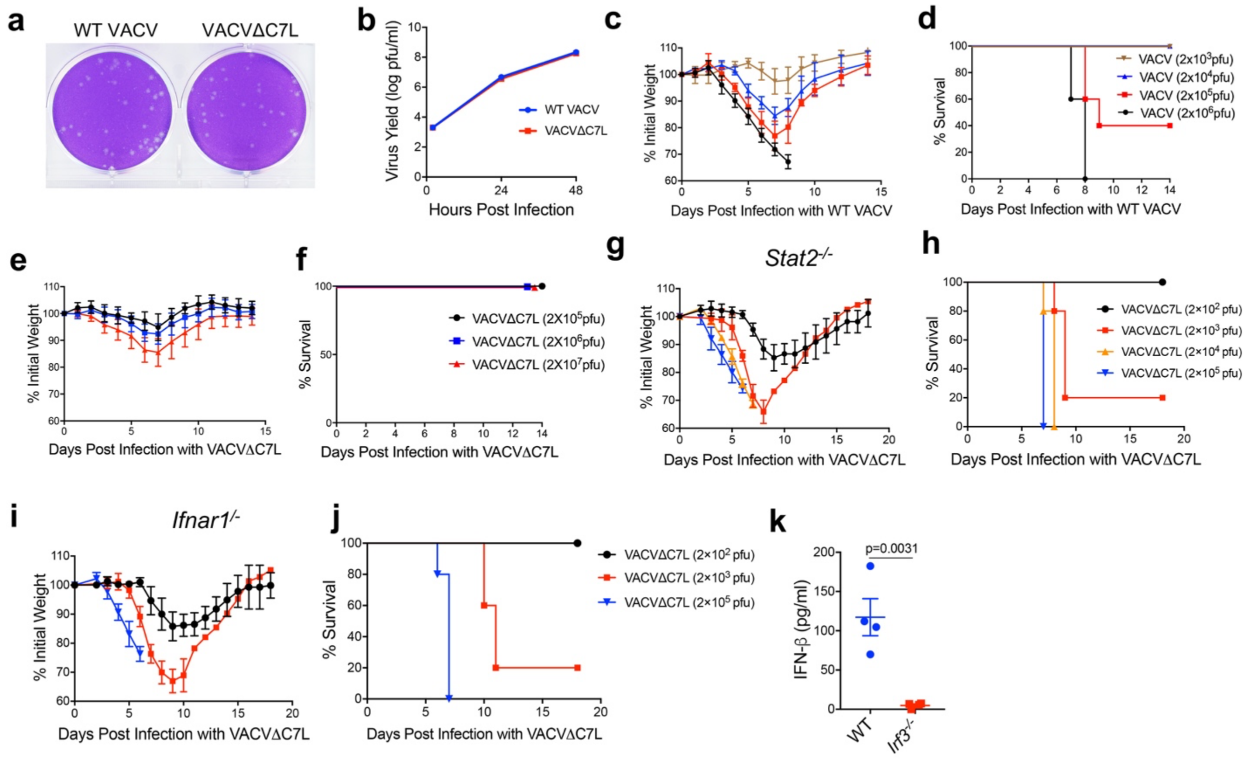

Supplementary Fig. 1 Determination of LD50 of WT VACV or VACVΔC7L intranasal infection in mice. (**a**) Viral plaques of WT VACV and VACVΔC7L at day 2 post infection in BSC40 cells. (**b**) Viral growth curve in BSC40 cells at an initial MOI of 0.05. (**c-d**) shown are the percentages of initial weight (**c**) or Kaplan-Meier survival curve (**d**) of WT C57BL/6J control mice (n=5 in each group) over days post intranasal infection with WT VACV at different doses. (**e-f**) shown are the percentages of initial weight (**e**) or Kaplan-Meier survival curve (**f**) of WT C57BL/6J control mice (n=5 in each group) over days post intranasal infection with VACVΔC7L at different doses. *Stat2*$^{-/-}$ or *Ifnar1*$^{-/-}$ mice were infected with VACVΔC7L at different doses. Mice were monitored for weight daily. (**g**) and (**i**) shown are the percentages of initial weight over days in *Stat2*$^{-/-}$ (**g**) or *Ifnar1*$^{-/-}$ mice (**i**) post intranasal infection with VACVΔC7L at increasing doses. (**h**) and (**j**) Kaplan-Meier survival curve of *Stat2*$^{-/-}$ (**h**) or *Ifnar1*$^{-/-}$ mice (**j**) infected with VACVΔC7L at increasing doses (n=5 in each group).
Data are representative of two independent experiments. (**k**) Levels of IFN-β in BAL from VACVΔC7L (2×10^7 pfu)-infected WT or *Irf3*$^{-/-}$ mice collected at day 3 post infection determined by ELISA. Two-tailed unpaired Student's t test was used for comparisons of two groups in the studies. Data are presented as mean ± SD. Source data are provided as a Source Data file.

Supplementary Fig. 2

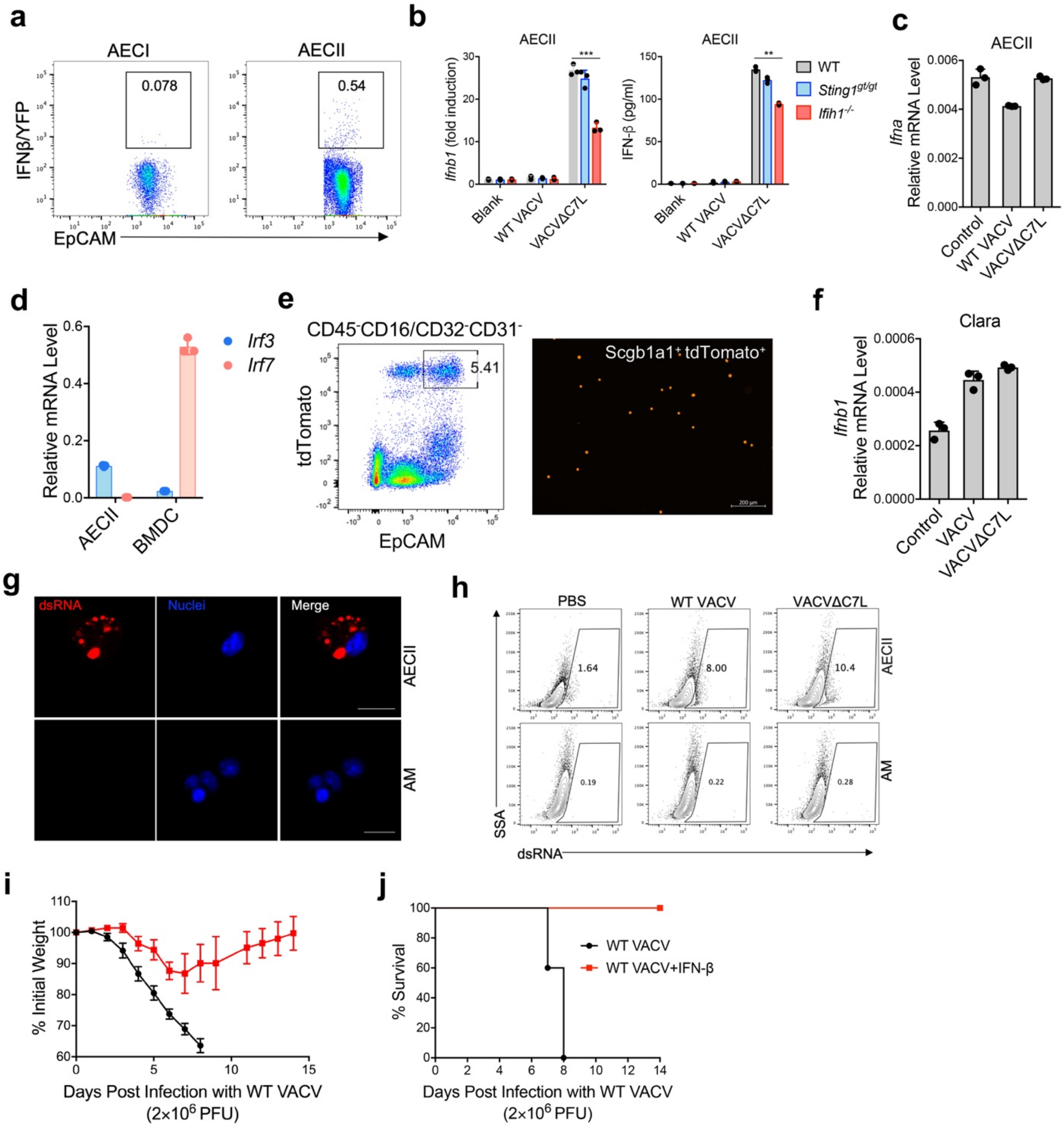

Supplementary Fig. 2 DsRNA was produced in AECIIs after VACVΔC7L infection and intranasal administration of IFN-β rescues mice from lethal WT VACV infection. (**a**) Dot plots showing percentages of IFN-β/YFP positive cells among AECI and AECII lung epithelial cells in VACVΔC7L-infected lungs from *Ifnb1*^{Eyfp} mice determined by FACS. (**b**) AECII were generated through culturing lineage negative epithelial progenitor cells isolated from WT, *Sting1*$^{gt/gt}$, or *Ifih1*$^{-/-}$ mice as described in Fig. 2. Left: RT-PCR analyses of *Ifnb1* gene expression of AECIIs infected with either WT VACV or VACVΔC7L at a MOI of 5 for 12 h. Right: ELISA analyses of IFN-β levels in the supernatants of AECIIs infected with either WT VACV or VACVΔC7L at a MOI of 5 for 24 h. (**c**) RT-PCR analyses of *Ifna* gene expression of WT AECIIs infected with either WT VACV or VACVΔC7L at a MOI of 5 for 12 h. (**d**) RT-PCR analyses of IRF3 and IRF7 gene expression in AECIIs or BMDC cells. GAPDH is used as internal control. (**e**) Left: Gating strategy for the isolation of Clara cells that are $CD45^-$ $CD16/CD32^-CD31^-EpCAM^+Scgb1a1^+$. Right: Representative images showing isolated Clara cells. Bar: 200 μm. (**f**) RT-PCR analyses of Ifnb1 gene expression of isolated Clara cells infected with either WT VACV or VACVΔC7L at a MOI of 5 for 12 h. (**g**) Representative confocal images showing dsRNA staining in AECIIs or AM cells infected with VACVΔC7L at a MOI of 5 for 16 h. Scale bar, 10 μm. (**h**) FACS analysis of dsRNA from AECIIs or AM cells infected with VACVΔC7L at a MOI of 5 for 16 h. C57BL/6J mice were infected with WT VACV at 2 x 10^6 pfu. They were either treated with intranasal administration of IFN-β (1 μg/mouse) or PBS at day one post infection. (**i-j**) shown are the percentages of initial weight (**i**) or Kaplan-Meier survival curve (**j**) over days. (n=5 in each group). ** $p<0.01$, and *** $p<0.001$ (unpaired t test). Data are representative of two (e and g) and represented as mean ± SD. Source data are provided as a Source Data file.

Supplementary Fig. 3

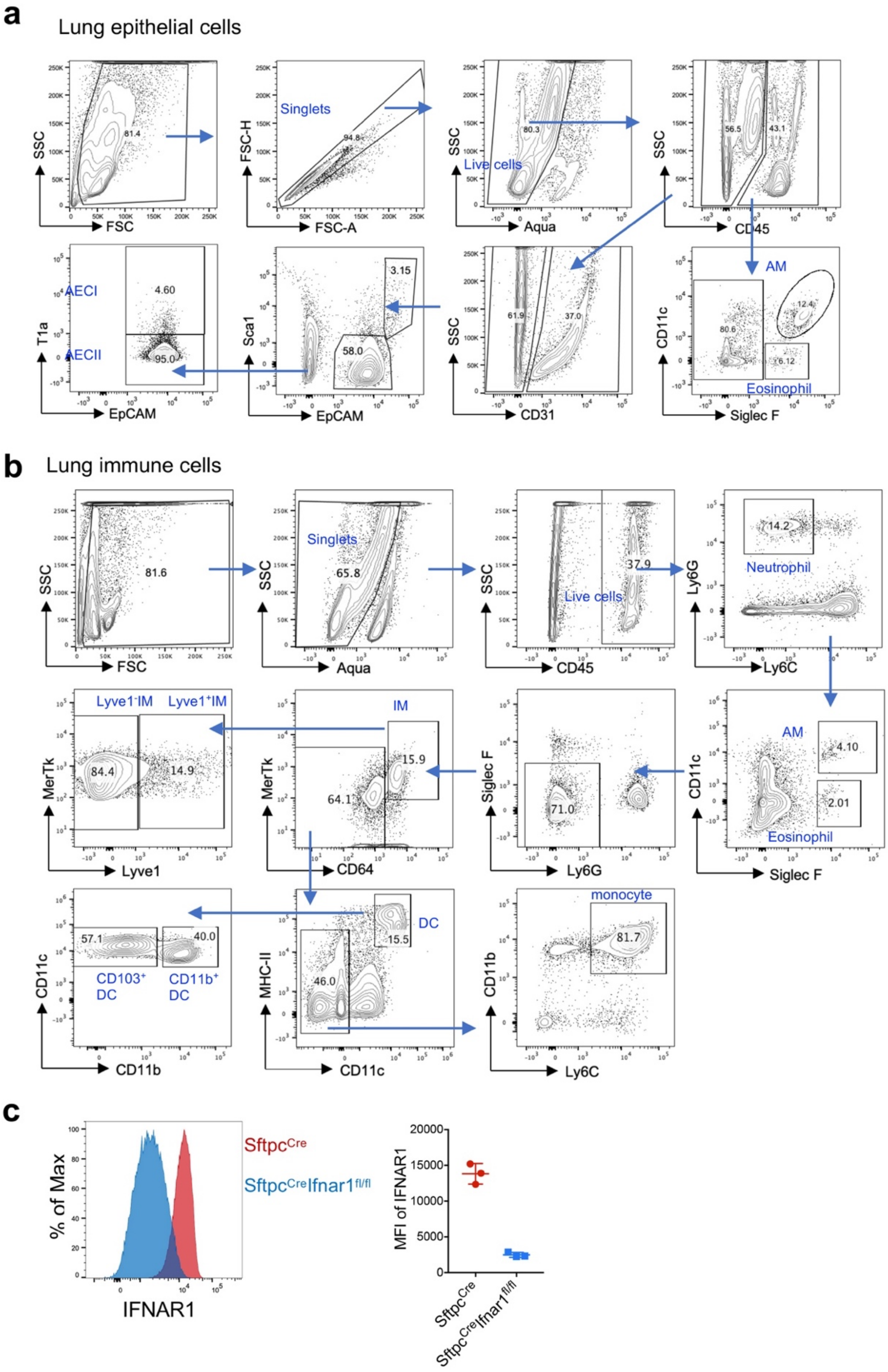

Supplementary Fig. 3 Gating strategy to define epithelial cells and myeloid cell populations in the lung. Within the single cell suspension, doublets and dead cells were excluded from analysis. (**a**) In $CD45^-$ cells, $CD31^+$ represents endothelial cells. In $CD31^-$, after excluding bronchioalveolar stem cells (BASCs), $EpCAM^+Sca1^-$ represents lung epithelial cells. AECIIs are further defined as $EpCAM^+T1a^-$ and AECIs are defined as $EpCAM^+T1a^+$. (**b**) In $CD45^+$ cells, Siglec F^+ $CD11c^+$ represents alveolar macrophages. Siglec F^+ $CD11c^-$ represents eosinophils. Neutrophils can be defined as $Ly6G^+$ $Ly6C^-$ cells. After excluding Siglec F^+, $Ly6G^+$, $CD3^+$, $CD49b^+$, $CD19^+$ cells, $CD64^+$ $MerTk^+$ cells represent IM and can be further divided into $Lyve1^+$ IM and $Lyve1^-$ IM. In $CD64^-MerTk^-$ cells, DCs are defined as MHC-II^+ $CD11c^+$ cells. Monocytes are further analyzed as $Ly6C^+$ $CD11b^+$ cells. (**c**) Left: Representative histogram of IFNAR1 expression in AECs from *Sftpc*^{Cre} and *Sftpc*^{Cre}*Ifnar*$^{fl/fl}$ mice after treatment of tamoxifen. Right: MFI of IFNAR1 expression in AECs from *Sftpc*^{Cre} and *Sftpc*^{Cre}*Ifnar*$^{fl/fl}$ mice after treatment of tamoxifen. Data are presented as mean ± SD. Source data are provided as a Source Data file.

Supplementary Fig. 4

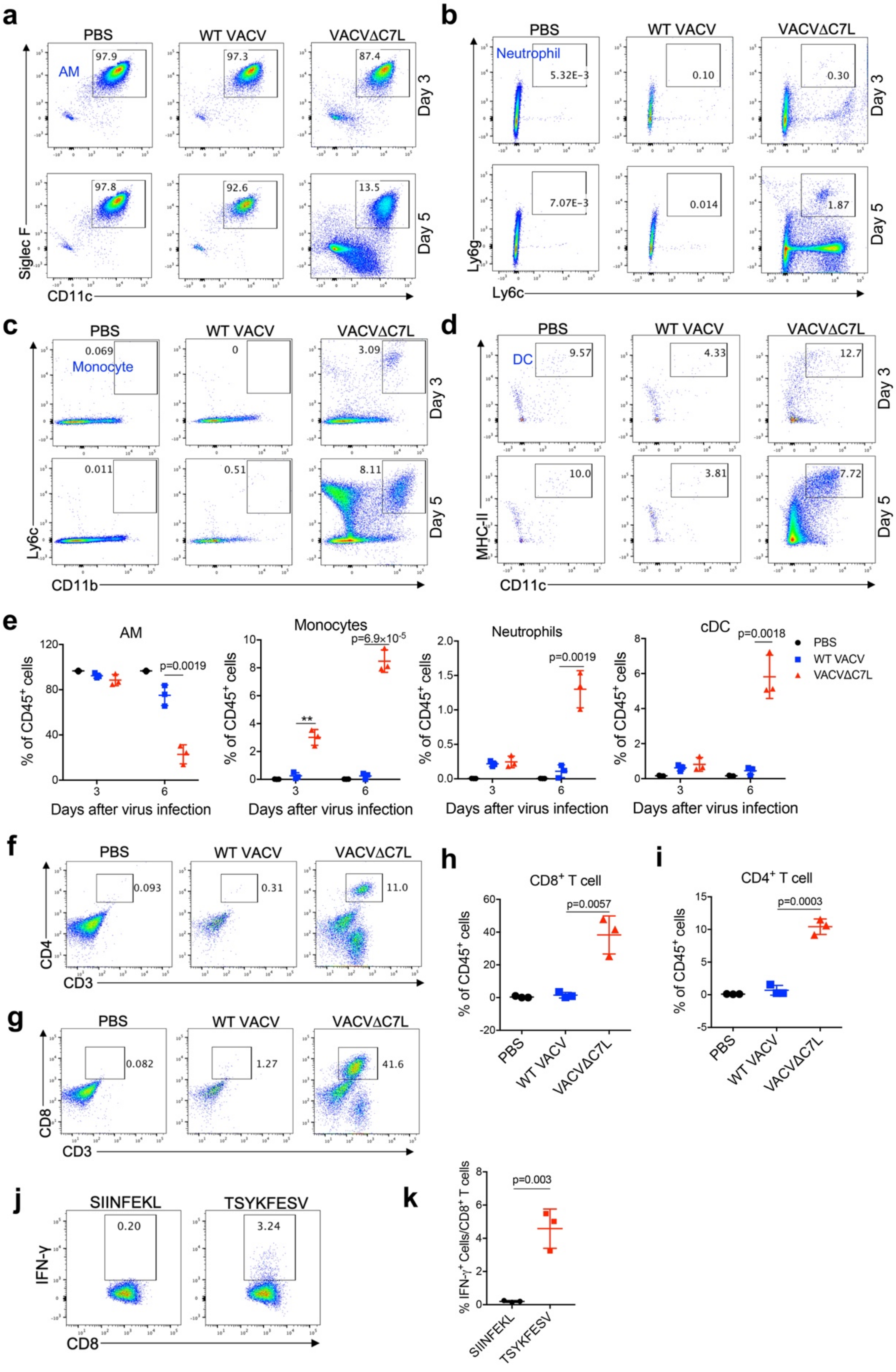

Supplementary Fig. 4 Intranasal infection of VACVΔC7L results in the influx of dendritic cells (DCs), monocytes, neutrophils, $CD8^+$, and $CD4^+$ T cells into bronchoalveolar space of the infected lungs. WT C57BL/6J mice were infected with either WT VACV at 2×10^5 pfu or with VACVΔC7L at 2×10^7 pfu, or mock-infected with PBS. BAL was collected at day 3 and day 5 post infection or PBS treatment. Myeloid cell populations in the BAL were analyzed by FACS. (**a**) Dot plots of Siglec F^+CD11c^+ lung alveolar macrophages in the BAL from mice infected with either WT VACV, VACVΔC7L, or mock infected. (**b**) Dot plots of $Ly6G^+Ly6C^+$ neutrophils in the BAL from mice infected with either WT VACV, VACVΔC7L, or mock infected. (**c**) Dot plots of $Ly6C^+CD11b^+$ inflammatory monocytes in the BAL from mice infected with either WT VACV, VACVΔC7L, or mock infected. (**d**) Dot plots of $MHCII^+CD11c^+$ DCs in the BAL from mice infected with either WT VACV, VACVΔC7L, or mock infected. (**e**) Quantification of data from (**a-d**) with independent experimental replicates. (**f-g**) Dot plots of $CD4^+$ or $CD8^+$ T cells in the BAL from mice at day 5 post infected with either WT VACV, VACVΔC7L, or mock infected. (**h-i**) Quantification of data from (**f-g**) with independent experimental replicates. (n=3 in each group). (**j**) Dot plots of B8R specific $CD8^+$ T cells in BAL from mice at day 5 post infected with VACVΔC7L. SIINFEKL as non-specific peptide control. (**k**) Quantification of data from **J**. Two-tailed unpaired Student's t test was used for comparisons of two groups in the studies. Data are representative of two (**f**-**k**) or three (**a**-**e**) independent experiments. Source data are provided as a Source Data file.

Supplementary Fig. 5

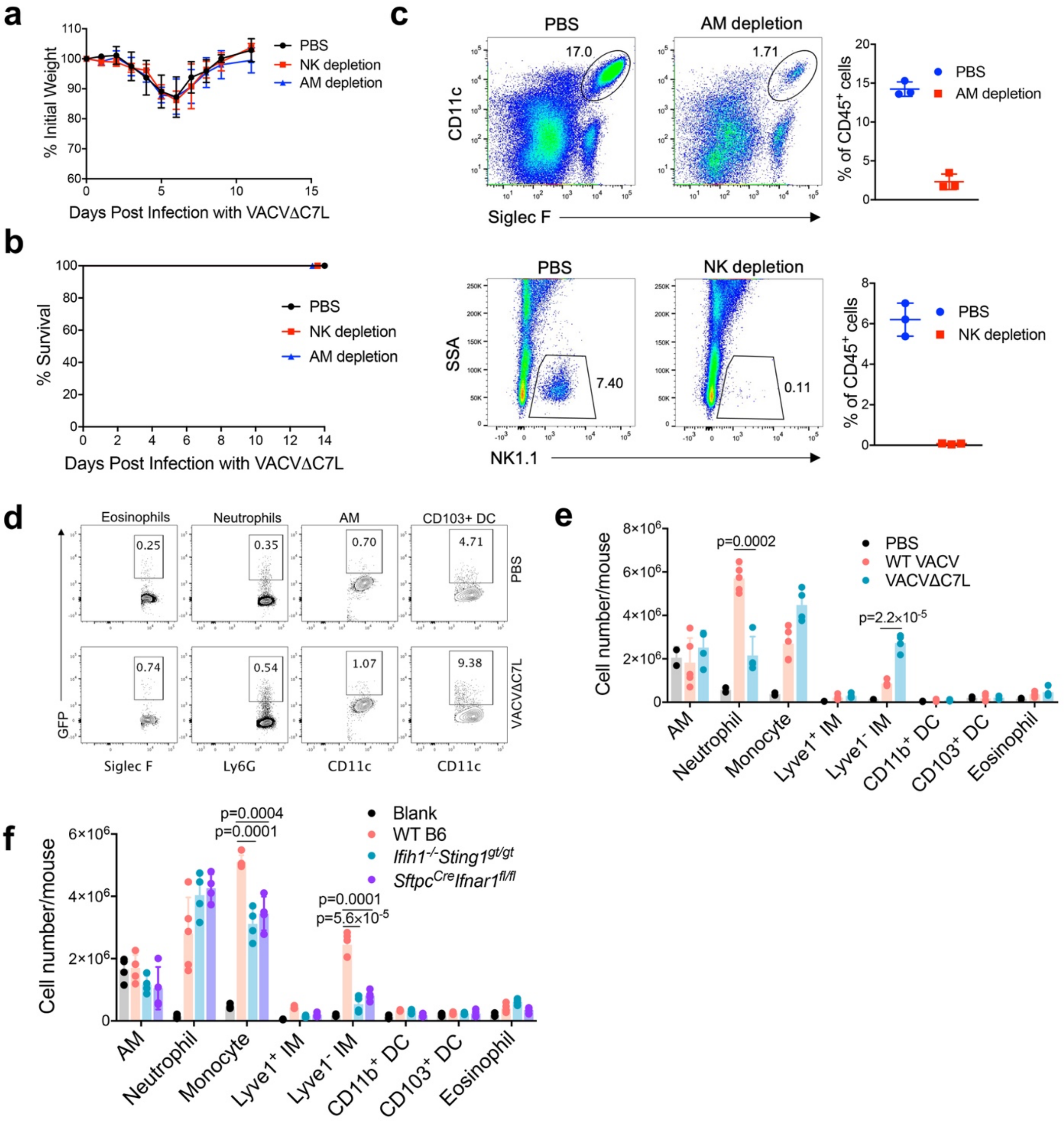

Supplementary Fig. 5 Alveolar macrophages and NK cells are not required for host defense against VACVΔC7L infection. (**a-b**) shown are the percentages of initial weight (**a**) or Kaplan-Meier survival curve (**b**) over days in WT C57BL/6J mice, NK cell depletion mice or AM depletion mice infected with VACVΔC7L at 2 x 10^7 pfu. (n=5 in each group). (**c**) Dot plot (Left) and bar graph (Right) showing AM and NK cells in the lungs after one day of second antibody or liposomes injections. (n=3 in each group). (**d**) Dot plots showing GFP^+ eosinophils, GFP^+ neutrophils, and GFP^+ AM and GFP^+ $CD103^+$ DCs in the lungs of *Ccr2-GFP* mice at day 3 post infection with VACVΔC7L at 2 x 10^7 pfu compared with PBS-mock infected mice. (**e**) Absolute cell number on changes of myeloid cells in the lungs of C57BL/6J mice at day 3 post infection with WT VACV, VACVΔC7L at 2 x 10^7 pfu or PBS-mock infected mice. (n=2-5 in each group). (**f**) Absolute cell number analysis on changes of myeloid cells in the lungs of WT C57BL/6J, $Ifih1^{-/-}Sting1^{gt/gt}$ or $Sftpc^{cre}Ifnar1^{fl/fl}$ mice at day 3 post infection with VACVΔC7L at 2 x 10^7 pfu. PBS was used as a mock infection blank control in WT mice. (n=4-5 in each group). Two-tailed unpaired Student's t test was used for comparisons of two groups in the studies. Data are presented as mean ± SD. Data are representative of two (**a-b, d**) or three (**e-f**) independent experiments. Source data are provided as a Source Data file.

Supplementary Fig. 6

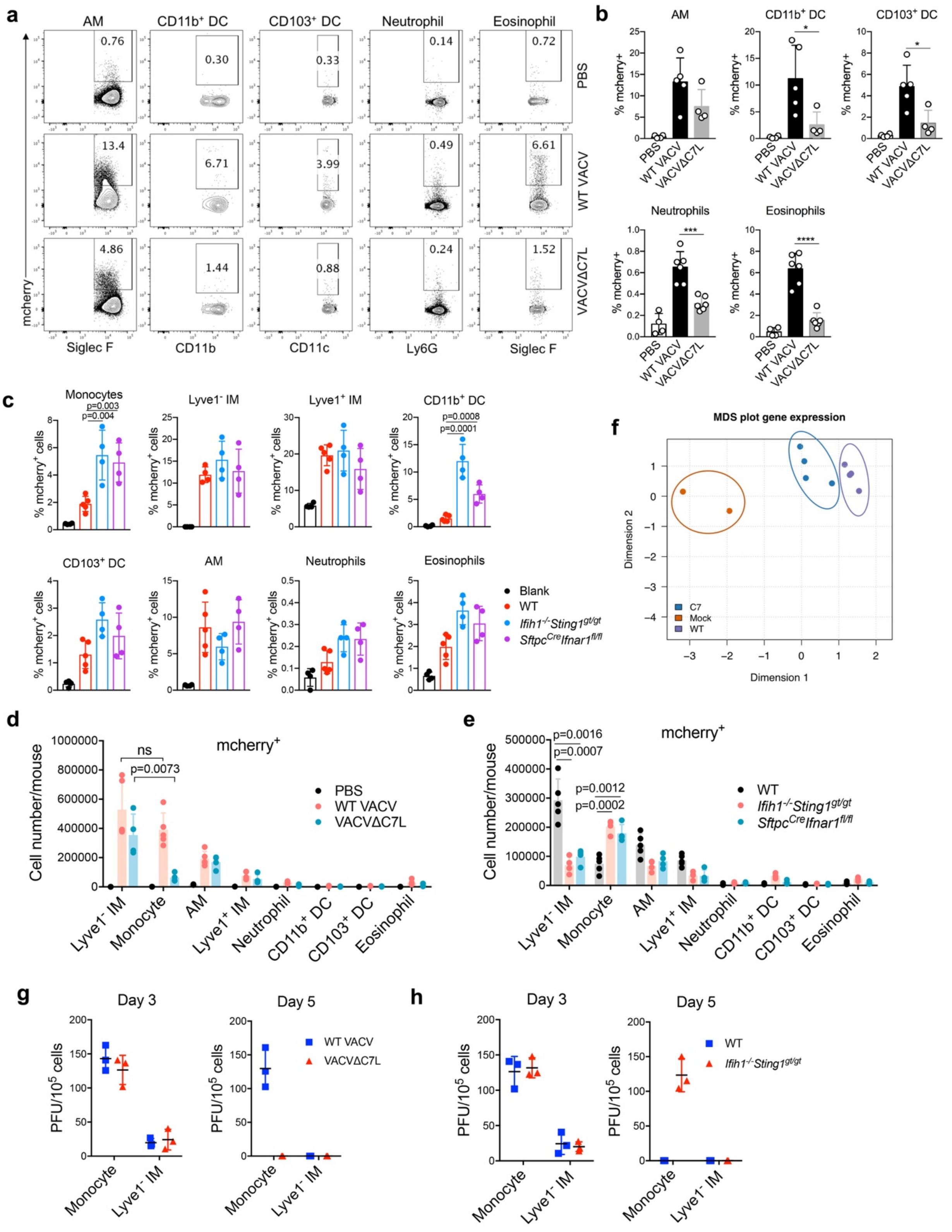

Supplementary Fig. 6 CCR2$^+$ inflammatory monocytes and IMs are the main cell populations infected by vaccinia virus. (**a-b**) Representative flow cytometry dot plots (**a**) and bar graph (**b**) showing mcherry$^+$ of different cell populations in the lungs of C57BL/6J mice at day 3 post infection with WT VACV-mcherry, VACVΔC7L-mcherry at 2 x 10^7 pfu or PBS-mock infected mice. (**c**) bar graph showing mcherry$^+$ of different cell populations in the lungs of C57BL/6J, *Ifih1*$^{-/-}$*Sting1*$^{gt/gt}$ or *Sftpc*^{cre}*Ifnar1*$^{fl/fl}$ mice at day 3 post infection with VACVΔC7L-mcherry at 2 x 10^7 pfu. PBS-mock infected C57BL/6J mice as blank control. (**d**) Bar graph showing absolute cell number of mcherry$^+$ cells in the lungs of C57BL/6J mice at day 3 post infection with WT VACV-mcherry, VACVΔC7L-mcherry at 2 x 10^7 pfu or PBS-mock infected mice. (**e**) Bar graph showing absolute cell number of mcherry$^+$ cells in the lungs of WT C57BL/6J, *Ifih1*$^{-/-}$*Sting1*$^{gt/gt}$ or *Sftpc*^{cre}*Ifnar1*$^{fl/fl}$ mice at day 3 post infection with VACVΔC7L-mcherry at 2 x 10^7 pfu. (**f**) Multidimensional scaling (MDS) plot of RNAseq results in monocytes based on host gene expression. (g) Virus titers in monocytes and Lyve1$^-$ IM isolated from WT mice infected with WT VACV, VACVΔC7L at 2 x 10^7 pfu for 3 or 5 days. (h) Virus titers in monocytes and Lyve1$^-$ IM isolated from WT or *Ifih1*$^{-/-}$*Sting1*$^{gt/gt}$ mice infected with WT VACV, VACVΔC7L at 2 x 10^7 pfu for 3 or 5 days. Two-tailed unpaired Student's t test was used for comparisons of two groups in the studies. Data are representative of one (**c**) or two (**a**-**b**, **d**) independent experiments and represented as mean ± SD. Source data are provided as a Source Data file.

Supplementary Table 1. List of primers used in this paper for qRT-PCR.

**Primer sequence**	SOURCE
qPCR *Ifnb1* For: TGGAGATGACGGAGAAGATG	Integrated DNA technologies IDT
qPCR *Ifnb1* Rev: TTGGATGGCAAAGGCAGT	
qPCR *Ifna* For: TCTGATGCAGCAGGTGGG	
qPCR *Ifna* Rev: AGGGCTCTCCAGACTTCTGCTCTG	
qPCR *Ccl4* For: GCCCTCTCTCTCCTCTTGCT	
qPCR *Ccl4* Rev: CTGGTCTCATAGTAATCCATC	
qPCR *Gapdh* For: AGGTCGGTGTGAACGGATTTG	
qPCR *Gapdh* Rev: TGTAGACCATGTAGTTGAGGTCA	
qPCR *Ccl5* For: GCCCACGTCAAGGAGTATTTCTA	
qPCR *Ccl5* Rev: ACACACTTGGCGGTTCCTTC	
qPCR *E5* For: TCTCGGACATTTCAGCCATC	
qPCR *E5* Rev: GGAAACATGTAAAGCAGCAGAG	
qPCR *A34* For: GGCATAGGAACATTTCTGCATTAC	
qPCR *A34* Rev: TACGACACTGATAAACCGCATT	
qPCR *A27* For: CCGTCCAGTCTGAACATCAAT	
qPCR *A27* Rev: GTGTTGTAAACGCAACGATGAA	
qPCR *F17* For: CTTCCTGCGTACTCAAAGTAGAT	
qPCR *F17* Rev: GTAGCATGTCCGTCCTCATAAA	
qPCR *Irf3* For: GTCTTAAGGAGCTGTTAGAGATGG	
qPCR *Irf3* Rev: TGGTCAGAGGTAAGGGAGATAG	
qPCR *Irf7* For: AAGACCAACTTCCGCTGTGC	
qPCR *Irf7* Rev: AGCATTGCTGAGGCTCACTT	
