# Supplementary material for: Lung type II alveolar epithelial cells collaborate with CCR2+ inflammatory monocytes in host defense against poxvirus infection
Source: Nat Commun. 2022 Mar 29;13:1671. doi: 10.1038/s41467-022-29308-2 (PMC8964745; doi:10.1038/s41467-022-29308-2)
Supplement: Supplementary file 2 — Reporting Summary [file 41467_2022_29308_MOESM2_ESM.pdf]

nature research

Corresponding author(s): Liang Deng

Last updated by author(s): Dec 31, 2021

Reporting Summary

Nature Research wishes to improve the reproducibility of the work that we publish. This form provides structure for consistency and transparency in reporting. For further information on Nature Research policies, see our Editorial Policies and the Editorial Policy Checklist.

Statistics

For all statistical analyses, confirm that the following items are present in the figure legend, table legend, main text, or Methods section.

n/a	Confirmed	
☐	☒	The exact sample size (n) for each experimental group/condition, given as a discrete number and unit of measurement
☐	☒	A statement on whether measurements were taken from distinct samples or whether the same sample was measured repeatedly
☐	☒	The statistical test(s) used AND whether they are one- or two-sided<br>*Only common tests should be described solely by name; describe more complex techniques in the Methods section.*
☐	☒	A description of all covariates tested
☐	☒	A description of any assumptions or corrections, such as tests of normality and adjustment for multiple comparisons
☐	☒	A full description of the statistical parameters including central tendency (e.g. means) or other basic estimates (e.g. regression coefficient) AND variation (e.g. standard deviation) or associated estimates of uncertainty (e.g. confidence intervals)
☐	☒	For null hypothesis testing, the test statistic (e.g. F, t, r) with confidence intervals, effect sizes, degrees of freedom and P value noted<br>*Give P values as exact values whenever suitable.*
☒	☐	For Bayesian analysis, information on the choice of priors and Markov chain Monte Carlo settings
☒	☐	For hierarchical and complex designs, identification of the appropriate level for tests and full reporting of outcomes
☒	☐	Estimates of effect sizes (e.g. Cohen's d, Pearson's r), indicating how they were calculated

Our web collection on statistics for biologists contains articles on many of the points above.

Software and code

Policy information about availability of computer code

Data collection	No software was used.
Data analysis	Flowjo 10.5.3 (Tree Star) for FACS results; GraphPad Prism 7 for statistics; Leica TCS SP8 and Image J (1.53) for imaging data analysis; ggplot2 (3.3.0) for Volcano plot.

For manuscripts utilizing custom algorithms or software that are central to the research but not yet described in published literature, software must be made available to editors and reviewers. We strongly encourage code deposition in a community repository (e.g. GitHub). See the Nature Research guidelines for submitting code & software for further information.

Data

Policy information about availability of data

All manuscripts must include a data availability statement. This statement should provide the following information, where applicable:

- Accession codes, unique identifiers, or web links for publicly available datasets
- A list of figures that have associated raw data
- A description of any restrictions on data availability

All data generated and supporting the findings of this study are available within this paper. RNA-seq data has been deposited in the NCBI Gene Expression Omnibus (GEO) database and are accessible through the GEO accession number: GSE158267. WT VACV transcripts are accessible through NCBI no. NC_006998.1.

Field-specific reporting

Please select the one below that is the best fit for your research. If you are not sure, read the appropriate sections before making your selection.

☒ Life sciences ☐ Behavioural & social sciences ☐ Ecological, evolutionary & environmental sciences

For a reference copy of the document with all sections, see nature.com/documents/nr-reporting-summary-flat.pdf

Life sciences study design

All studies must disclose on these points even when the disclosure is negative.

Sample size	Sample sizes were not predetermined and are indicated in the figure legends. The group sizes of mice and samples were chosen based on our experience with similar studies, common practice in this field and resource availability.
Data exclusions	We did not exclude data in this study.
Replication	All the experimental findings were reliably reproduced as validated by at least two independent experiments.
Randomization	Samples were randomized into experimental or control groups. Animals were randomized into different treatment groups.
Blinding	The investigator for viral titer determination was blinded. Investigators were not blinded to group allocation during data collection and/or analysis in other experiments, because the same researcher performed the experiment and analyzed the data.

Reporting for specific materials, systems and methods

We require information from authors about some types of materials, experimental systems and methods used in many studies. Here, indicate whether each material, system or method listed is relevant to your study. If you are not sure if a list item applies to your research, read the appropriate section before selecting a response.

Materials & experimental systems

n/a	Involved in the study	
☐	☒	Antibodies
☐	☒	Eukaryotic cell lines
☒	☐	Palaeontology and archaeology
☐	☒	Animals and other organisms
☒	☐	Human research participants
☒	☐	Clinical data
☒	☐	Dual use research of concern

Methods

n/a	Involved in the study	
☒	☐	ChIP-seq
☐	☒	Flow cytometry
☒	☐	MRI-based neuroimaging

Antibodies

Antibodies used	The following antibodies were used for flow cytometry: BioLegend: CD45.2 (104) Cat# 109822, CD45 (30-F11) Cat# 103112, CD45.1 (A20) Cat# 110722, EpCAM (G8.8) Cat# 118218, CD31 (MEC13.3) Cat# 102510, CD104 (346-11A) Cat# 123606, Ly6G (1A8) Cat# 127618. CD11c (N418) Cat# 117320. CD11b (M1/70) Cat# 101226, MHC II (M5/114.15.2) Cat# 107645, CD64 (X54-5/7.1) Cat# 139323, CD3e (145-2C11) Cat# 100341, CD4 (GK1.5) Cat# 100428, CD8 (53-5.8) Cat# 140418, IFN-r (XMG1.2) Cat# 505810, IFNAR-1 (MAR1-5A3) Cat# 127306, T1a (8.1.1) Cat# 127408. BD Biosciences: CD45 (30-F11) Cat# 564279, Siglec F (E50-2440) Cat# 562680, CD19 (1D3) Cat# 562701, CD49b (DX5) Cat# 563063. Thermo Fisher: CD16/CD32 (93) Cat# 13-0161-82, Ly6C (HK1.4) Cat# 45-5932-82, MerTk (DS5MMER) Cat# 53-5751-82, Lyve1 (ALY7) Cat# 12-0443-82. All antibodies are used at 1:200.<br>The following antibodies were used for imaging: anti-GFP (Abcam ab13970), anti-SP-C (Millipore AB3786), anti-C7 (this paper, 1:500), anti-dsRNA (Millipore MABE1134, clone: rJ2, 1:100). Goat anti-Chicken IgY (H+L) Secondary Antibody, Alexa Fluor 488 (ThermoFisher A-11039), Goat anti-Rabbit IgG (H+L) Cross-Adsorbed Secondary Antibody, Alexa Fluor 488 (ThermoFisher A-11008), Goat anti-Rabbit IgG (H+L) Cross-Adsorbed Secondary Antibody, Alexa Fluor 594 (ThermoFisher A-11012), Goat anti-Mouse IgG (H+L) Highly Cross-Adsorbed Secondary Antibody, Alexa Fluor 594 (ThermoFisher A-11032). All antibodies are used at 1:1000 unless otherwise noted.<br>The following antibodies were used for western blot: anti-phospho-IRF3 (CST, 4947, clone: 4D4G, 1:1000), anti-IRF3 (CST, 4302, clone: D83B9, 1:1000), anti-C7 antibody (this paper, 1:500), anti-FLAG (Sigma, F3165, clone: M2, 1:1000), anti-β-actin (CST, 4967, 1:2000), anti-GAPDH (CST, 2118, clone: 14C10, 1:2000) and Anti-rabbit or mouse HRP-linked IgG antibody (CST 7074 or 7076, 1:5000).
Validation	The specificities of listed FACS antibodies have been validated by the manufacturer by flow cytometry.<br>CD45.2 (104) Cat# 109822: https://www.biolegend.com/en-us/products/alexa-fluor-700-anti-mouse-cd45-2-antibody-3393?GroupID=BLG1934<br>CD45 (30-F11) Cat# 103112: https://www.biolegend.com/en-us/products/apc-anti-mouse-cd45-antibody-97?GroupID=BLG6837<br>CD45.1 (A20) Cat# 110722: https://www.biolegend.com/en-us/products/pacific-blue-anti-mouse-cd45-1-antibody-3105<br>EpCAM (G8.8) Cat# 118218: https://www.biolegend.com/en-us/products/apc-cyanine7-anti-mouse-cd326-ep-cam-antibody-5577?

GroupID=BLG5748
CD31 (MEC13.3) Cat# 102510: https://www.biolegend.com/de-de/products/apc-anti-mouse-cd31-antibody-375
CD104 (346-11A) Cat# 123606: https://www.biolegend.com/en-us/products/fitc-anti-mouse-cd104-antibody-4491?GroupID=BLG5353
Ly6G (1A8) Cat# 127618: https://www.biolegend.com/en-us/search-results/pe-cyanine7-anti-mouse-ly-6g-antibody-6139?GroupID=BLG7234
CD11c (N418) Cat# 117320: https://www.biolegend.com/en-us/products/alexa-fluor-700-anti-mouse-cd11c-antibody-3429?GroupID=BLG11937
CD11b (M1/70) Cat# 101226: https://www.biolegend.com/en-us/products/apc-cyanine7-anti-mouse-human-cd11b-antibody-3930?GroupID=BLG10616
MHC II (M5/114.15.2) Cat# 107645: https://www.biolegend.com/fr-ch/search-results/brilliant-violet-785-anti-mouse-i-a-i-e-antibody-12087
CD64 (X54-5/7.1) Cat# 139323: https://www.biolegend.com/en-us/products/brilliant-violet-605-anti-mouse-cd64-fcgammari-antibody-13611?GroupID=BLG8806
CD3e (145-2C11) Cat# 100341: https://www.biolegend.com/en-us/products/brilliant-violet-421-anti-mouse-cd3epsilon-antibody-7132?GroupID=BLG6744
CD4 (GK1.5) Cat# 100428: https://www.biolegend.com/en-us/products/pacific-blue-anti-mouse-cd4-antibody-3316?GroupID=BLG4745
CD8 (53-5.8) Cat# 140418: https://www.biolegend.com/en-us/products/percp-cyanine5-5-anti-mouse-cd8b2-antibody-17484?GroupID=BLG8876
IFN-r (XMG1.2) Cat# 505810: https://www.biolegend.com/en-us/products/apc-anti-mouse-ifn-gamma-antibody-993?GroupID=GROUP24
IFNAR-1 (MAR1-5A3) Cat# 127306: https://www.biolegend.com/en-us/products/biotin-anti-mouse-ifnar-1-antibody-4782?GroupID=BLG5784
T1a (8.1.1) Cat# 127408: https://www.biolegend.com/en-us/search-results/pe-anti-mouse-podoplanin-antibody-4882?GroupID=BLG5772
CD45 (30-F11) Cat# 564279: https://www.bdbiosciences.com/en-us/products/reagents/flow-cytometry-reagents/research-reagents/single-color-antibodies-ruo/buv395-rat-anti-mouse-cd45.564279
Siglec F (E50-2440) Cat# 562680: https://www.bdbiosciences.com/en-us/products/reagents/flow-cytometry-reagents/research-reagents/single-color-antibodies-ruo/alexa-fluor-647-rat-anti-mouse-siglec-f.562680
CD19 (1D3) Cat# 562701: https://www.bdbiosciences.com/en-us/products/reagents/flow-cytometry-reagents/research-reagents/single-color-antibodies-ruo/bv421-rat-anti-mouse-cd19.562701
CD49b (DX5) Cat# 563063: https://www.bdbiosciences.com/en-us/products/reagents/flow-cytometry-reagents/research-reagents/single-color-antibodies-ruo/bv421-rat-anti-mouse-cd49b.563063
CD16/CD32 (93) Cat# 13-0161-82: https://www.thermofisher.com/antibody/product/CD16-CD32-Antibody-clone-93-Monoclonal/13-0161-82
Ly6C (HK1.4) Cat# 45-5932-82: https://www.thermofisher.com/antibody/product/Ly-6C-Antibody-clone-HK1-4-Monoclonal/45-5932-82
MerTk (DS5MMER) Cat# 53-5751-82: https://www.thermofisher.com/antibody/product/MERTK-Antibody-clone-DS5MMER-Monoclonal/53-5751-82
Lyve1 (ALY7) Cat# 12-0443-82: https://www.thermofisher.com/antibody/product/LYVE1-Antibody-clone-ALY7-Monoclonal/12-0443-82
The specificities of listed imaging antibodies have been validated by the manufacturer by immunofluorescence.
anti-GFP (Abcam ab13970): https://www.abcam.com/gfp-antibody-ab13970.html
anti-SP-C (Millipore AB3786): https://www.emdmillipore.com/US/en/product/Anti-Prosurfactant-Protein-C-proSP-C-Antibody,MM_NF-AB3786
anti-dsRNA (Millipore MABE1134): https://www.emdmillipore.com/US/en/product/Anti-dsRNA-Antibody-clone-rJ2,MM_NF-MABE1134-100UL
Goat anti-Chicken IgY (H+L) Secondary Antibody, Alexa Fluor 488: https://www.thermofisher.com/antibody/product/Goat-anti-Chicken-IgY-H-L-Secondary-Antibody-Polyclonal/A-11039
Goat anti-Rabbit IgG (H+L) Cross-Adsorbed Secondary Antibody, Alexa Fluor 488: https://www.thermofisher.com/antibody/product/Goat-anti-Rabbit-IgG-H-L-Cross-Adsorbed-Secondary-Antibody-Polyclonal/A-11008
Goat anti-Rabbit IgG (H+L) Cross-Adsorbed Secondary Antibody, Alexa Fluor 594: https://www.thermofisher.com/antibody/product/Goat-anti-Rabbit-IgG-H-L-Cross-Adsorbed-Secondary-Antibody-Polyclonal/A-11012
Goat anti-Mouse IgG (H+L) Highly Cross-Adsorbed Secondary Antibody, Alexa Fluor 594: https://www.thermofisher.com/antibody/product/Goat-anti-Mouse-IgG-H-L-Highly-Cross-Adsorbed-Secondary-Antibody-Polyclonal/A-11032
The specificities of listed WB antibodies have been validated by the manufacturer by western blot.
anti-phospho-IRF3 (CST, 4947, clone: 4D4G): https://www.cellsignal.com/products/primary-antibodies/phospho-irf-3-ser396-4d4g-rabbit-mab/4947
anti-IRF3 (CST, 4302, clone: D83B9): https://www.cellsignal.com/products/primary-antibodies/irf-3-d83b9-rabbit-mab/4302
anti-FLAG (Sigma, F3165, clone: M2): https://www.sigmaaldrich.com/US/en/product/sigma/f3165
anti-β-actin (CST, 4967): https://www.cellsignal.com/products/primary-antibodies/b-actin-antibody/4967
anti-GAPDH (CST, 2118, clone: 14C10): https://www.cellsignal.com/products/primary-antibodies/gapdh-14c10-rabbit-mab/2118
Anti-rabbit HRP-linked IgG antibody (CST 7074): https://www.cellsignal.com/products/secondary-antibodies/anti-rabbit-igg-hrp-linked-antibody/7074
Anti-mouse HRP-linked IgG antibody (CST 7076): https://www.cellsignal.com/products/secondary-antibodies/anti-mouse-igg-hrp-linked-antibody/7076

Eukaryotic cell lines

Policy information about cell lines

Cell line source(s)	BSC40, HEK293T, RAW264.7, BHK21, HeLa and THP-1 cell lines were purchased from ATCC.

Authentication | Cell lines were not authenticated.

Mycoplasma contamination | All of the cell lines were tested negative for mycoplasma contamination.

Commonly misidentified lines (See ICLAC register) | No commonly misidentified cell lines were used.

Animals and other organisms

Policy information about studies involving animals; ARRIVE guidelines recommended for reporting animal research

Laboratory animals

C57BL/6, IFNb/YFP reporter mouse, cGAS-/-, STAT2-/-, IFNAR1-/-, Sftpc-CreERT2, Ifnarfl/fl, Scgb1a1-CreERTM, Rosa26-lox-stop-lox-TdTomato mice were purchased from the Jackson Laboratory. STINGGt/Gt mice were generated in the laboratory of Russell Vance (University of California, Berkeley). MDA5-/- mice were generated in Marco Colonna's laboratory (Washington University). Irf3-/- mice were provided by Ruslan Medzhitov (Yale University). MDA5-/-STING Gt/Gt, Scgb1a1CreR26tdT and SftpccreIfnar1fl/fl mice were breeded in our lab. CCR2-GFP and CCR2-DTR mice were provided by Eric Pamer (University of Chicago). Female mice between 6-8 weeks old were used. These mice were maintained in the animal facility at the Sloan Kettering Cancer Institute. The holding room was maintained at 12:12-h light:dark cycle with room temperature of 72 +/-2 F and relatively humidity ranging from 30%-70%.

Wild animals

The study did not involve wild animals.

Field-collected samples

The study did not involve samples collected from the field.

Ethics oversight

All procedures were performed in strict accordance with the recommendations in the Guide for the Care and Use of Laboratory Animals of the National Institute of Health. The protocol was approved by the Committee on the Ethics of Animal Experiments of Sloan-Kettering Cancer Institute.

Note that full information on the approval of the study protocol must also be provided in the manuscript.

Flow Cytometry

Plots

Confirm that:

☒ The axis labels state the marker and fluorochrome used (e.g. CD4-FITC).

☒ The axis scales are clearly visible. Include numbers along axes only for bottom left plot of group (a 'group' is an analysis of identical markers).

☒ All plots are contour plots with outliers or pseudocolor plots.

☒ A numerical value for number of cells or percentage (with statistics) is provided.

Methodology

Sample preparation

To measure viral titers within different organs, lungs, livers, brains and spleens were harvested, placed into tubes with 1 ml of PBS, and homogenized using the Miltenyi GentleMACS™ Dissociator. Blood were collected into 1.5 ml eppendorf tubes and serum were kept for after centrifigation.To prepare single cell suspension from the BALF and lungs, bronchoalveolar lavage fluid (BALF) was harvested with intratracheal infusion of 1 ml of cold PBS. To harvest lungs, lungs were cleared of blood with perfusion of cold PBS through the right ventricle. The lung lobes were excised and digested with Collagenase D (2 mg/ml) and DNase I (100 μg/ml) for 45 mins at 37°C. After dissociation of the lung tissue in the Miltenyi GentleMACS™ Dissociator, lung homogenates were incubated with red blood cell lysis buffer on ice for 5 min and then quenched with cold PBS. The cell pellets were resuspended with MACS buffer (Miltenyi Biotec) to generate single cell suspension and then filtered through 70 μm nylon mesh prior to FACS analysis.

Instrument

LSR Fortessa (BD Biosciences)

Software

Flowjo 10.5.3 (Tree Star)

Cell population abundance

When cells were sorted or enriched, the purity was confirmed by flow cytometry and in each case was above 90% purity.

Gating strategy

Cells were first gated by FSC/SSC. Singlets were gated according to the pattern of FSC-H vs. FSC-A. Positive populations were determined by the specific antibodies, which were distinct from negative populations.

☒ Tick this box to confirm that a figure exemplifying the gating strategy is provided in the Supplementary Information.
